# Reusable Polyphenol Building Blocks Enable Recyclable Epoxy‐Amine Thermosets via Methanolysis

**DOI:** 10.1002/anie.3352626

**Published:** 2026-09-16

**Authors:** Yi‐Min Tu, Yuhang Jiang, Kun Li, Qing Cao, Yizhe Pan, Christoph Tzschucke, Andreas J. Achazi, Rainer Haag

**Affiliations:** ^1^ Institute of Chemistry and Biochemistry Freie Universität Berlin Berlin Germany; ^2^ National Engineering Laboratory of Eco‐Friendly Polymeric Materials (Sichuan), State Key Laboratory of Advanced Polymer Materials, College of Chemistry Sichuan University Chengdu P. R. China; ^3^ IT and Data Services Zuse Institute Berlin Berlin Germany

**Keywords:** antibacterial materials, biobased epoxy, building blocks, methanolysis, thermosets

## Abstract

Biobased polyphenols represent attractive alternatives to bisphenol A for epoxy thermosets, yet most bio‐derived epoxy networks remain permanently cross‐linked and are difficult to recycle. Herein, we report a modular molecular design strategy that converts renewable polyphenols into reusable functional building blocks for recyclable epoxy–amine thermosets. Distinct from conventional degradable epoxy networks, this platform enables systematic structural variation and establishes a clear structure–depolymerization relationship. Kinetic and computational analyses further support a previously unrecognized ether‐assisted methanolysis mechanism, in which neighboring ether functionalities and a favorable aliphatic amine environment lower the apparent kinetic barrier and modulate the local methanol environment around the cleavable ester bonds in a configuration‐dependent manner. Consequently, the resulting thermosets undergo efficient methanolysis under mild conditions (70°C), affording reusable molecular components while maintaining tunable thermal and mechanical properties. Furthermore, carbon fiber–reinforced composites based on the optimized formulation can be efficiently depolymerized, yielding intact fibers and reusable building blocks in 88% isolated yield. The recovered polyols can be further upcycled into antibacterial quaternary ammonium materials, demonstrating a value‐added reuse pathway. This work identifies a previously unrecognized molecular promotion mechanism for catalyst‐free methanolysis and translates this mechanism insight into a structural design principle for recyclable polyphenol‐based epoxy–amine thermosets.

## Graphical abstract

A biobased epoxy–amine platform was developed using functional building blocks derived from polyphenols. These thermosets exhibited tunable mechanical and thermal properties, together with efficient, catalyst‐free chemical recycling via methanolysis. Beyond the recovery of reusable building blocks, the recycled polyols could be further upcycled into antibacterial materials. This work provides guiding principles for the design of structurally tunable and recyclable epoxy–amine thermosets.

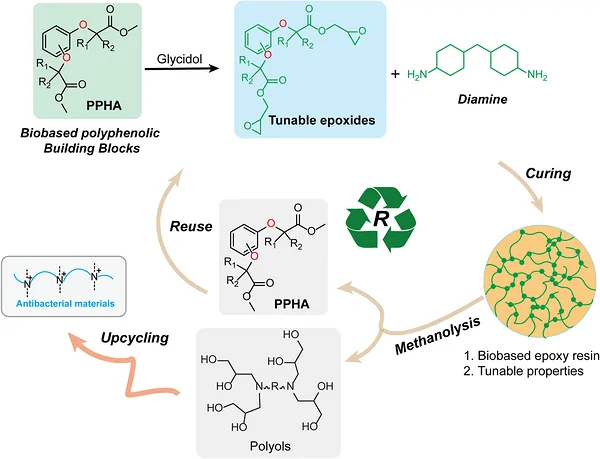

## 1 Introduction

Polyphenols constitute a central class of aromatic building blocks for high‐performance epoxy thermosets, as their rigid aromatic frameworks and multiple phenolic functionalities enable high cross‐linking density and excellent thermal and mechanical properties. Among them, bisphenol A (BPA) has long served as the most widely used polyphenolic precursor for epoxy–amine resins, forming the backbone of commercial epoxy formulations for structural composites [1, 2, 3, 4, 5]. Owing to their well‐established performance, BPA‐based epoxies cured with diverse amines continue to dominate the market for load‐bearing applications, including automotive components and wind‐turbine blades [6, 7, 8, 9, 10, 11, 12].

Despite their outstanding performance, BPA‐based epoxy thermosets rely on fossil‐derived feedstocks and raise concerns over the potential health impacts of BPA, leading to significant sustainability and end‐of‐life challenges [13, 14, 15, 16]. These limitations have driven increasing interest in bio‐based polyphenols derived from renewable resources as potential alternatives to BPA. Owing to their structural similarity to BPA, many bio‐based polyphenols can be readily integrated into conventional epoxy curing chemistries, enabling the construction of high‐performance thermosets. Recent studies have demonstrated that precise molecular and supramolecular design can effectively regulate polymer and epoxy‐resin properties. Long‐range ordered block‐copolymer structures have been employed to construct efficient phonon‐transport pathways, while liquid‐crystalline epoxy architectures, fluorinated semi‐interpenetrating networks, and tailored flexible spacers have been used to improve thermal conductivity, dielectric properties, and thermomechanical performance [17, 18, 19]. These studies establish important structure–property relationships for advanced thermal‐management materials but do not primarily address chemically recyclable epoxy–amine networks. Therefore, integrating high‐performance polyphenol‐derived epoxy architectures with well‐defined chemical recyclability and reusable degradation products remains a key challenge.

Nevertheless, replacing BPA with bio‐based polyphenols alone does not inherently address recyclability. Similar to BPA‐based systems, bio‐based polyphenol‐derived epoxy thermosets typically form permanently cross‐linked and chemically resistant networks, leading to end‐of‐life recycling pathways that largely mirror those of conventional epoxies and remain mainly limited to low‐value mechanical recycling or disposal by incineration and landfill (Figure 1a) [5, 20, 21, 22, 23, 24]. In epoxy‐based composites, the strong interfacial adhesion between the epoxy matrix and reinforcing carbon fibers therefore remains a major obstacle to the high‐value recovery of both polymer matrices and fibers.

**FIGURE 1:**
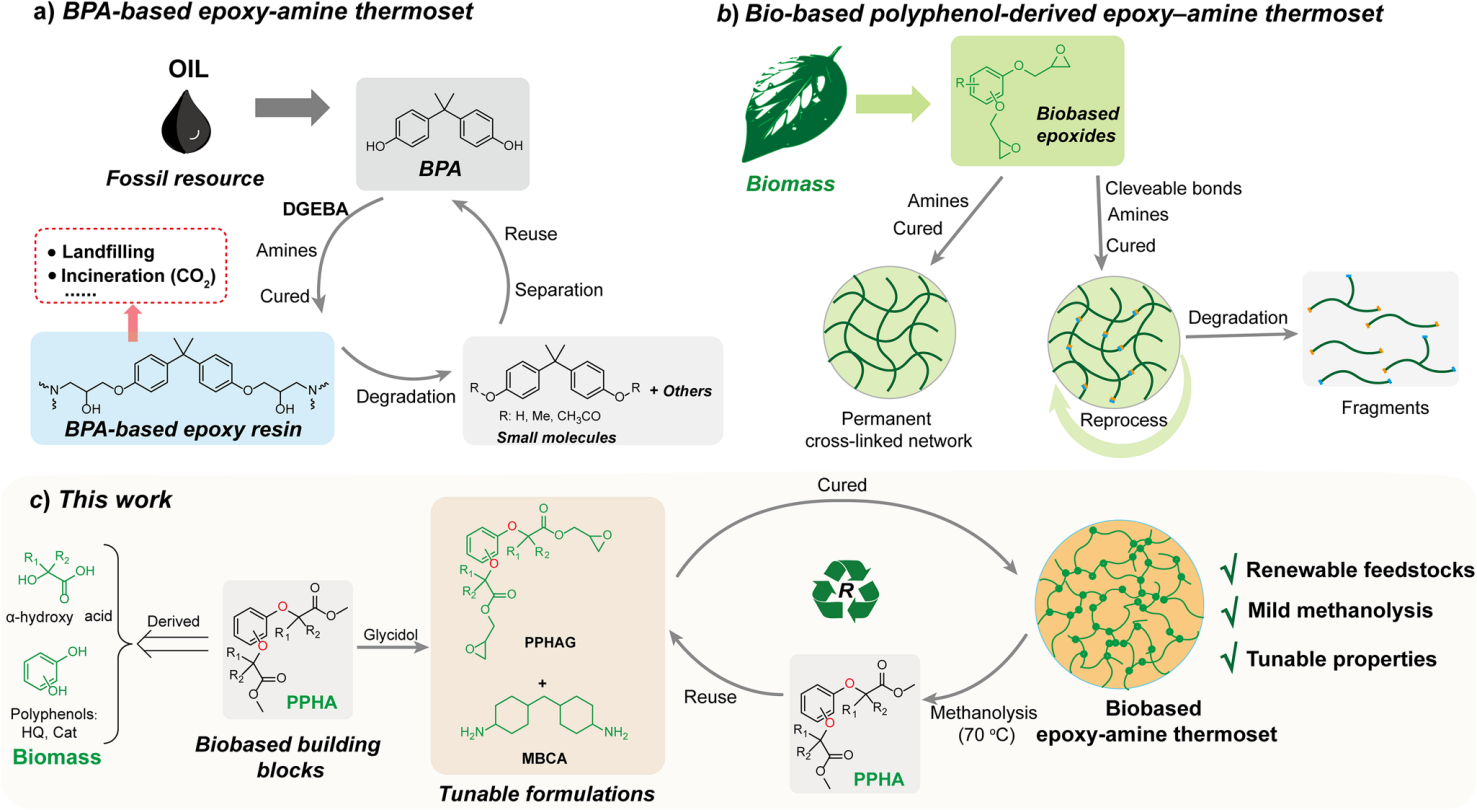
(a) The life cycle of BPA‐based epoxy‐amine thermoset. (b) Life cycle of epoxy‐amine thermoset derived from biobased polyphenols. (c) This work: design of biobased building blocks (derived from hydroquinone, catechol, and α‐hydroxy acid) for epoxy–amine systems with methanolysis‐enabled recovery and reuse of PPHA.

To overcome the intrinsic constraint, chemical recycling has attracted growing interest as an effective strategy to selectively cleave thermoset networks and recover reusable small molecules and reinforcing fibers from epoxy composites (Figure 1a) [25, 26, 27, 28, 29, 30, 31, 32, 33, 34, 35, 36]. By targeting covalent linkages formed during epoxy curing, such approaches have enabled the isolation of BPA and, in some cases, intact carbon fibers, as demonstrated by metal‐catalyzed [34, 35, 37], Lewis‐acid mediated [38], and solvolytic strategies [39]. Nevertheless, most reported chemical recycling methods rely on complex catalytic systems, harsh reaction conditions, or multistep processes, and predominantly focus on BPA recovery while generating mixed products.

Alternatively, an effective strategy is to introduce various cleavable linkages (e.g., ester [40, 41, 42, 43, 44], acetal [45], imine [46], disulfide [47, 48], and other dynamic covalent motifs [49, 50, 51, 52]) into thermosets to enable degradation and reprocessability. For example, recent studies [52, 53, 54, 55] have demonstrated recyclable high‐performance epoxy composites by incorporating cleavable motifs into epoxy networks, highlighting the potential of molecularly engineered thermosets for balancing mechanical robustness and recyclability. While such strategies enable composite recycling, especially fiber recovery, the degraded thermoset matrices typically yield structurally complex and chemically diverse fragments, limiting high‐value reuse (Figure 1b). A representative furan‐based epoxy–amine system reported by Wu and co‐workers [56] undergoes selective ester‐bond cleavage through mild methanolysis at 70°C, assisted by intrinsic hydrogen‐bond interactions involving the furan moieties in the epoxy network. However, because this behavior is closely associated with a specific furan‐based architecture, its structural tunability and broader applicability across different epoxy systems remain to be further established.

Herein, we develop a modular platform that converts biobased polyphenols and α‐hydroxy acids into structurally tunable PPHA building blocks for recyclable epoxy–amine thermosets (Figure 1c). Their substitution position, steric environment, and neighboring ether functionality can be systematically varied, enabling direct correlations between molecular structure and methanolysis behavior. We hypothesized that the neighboring ether functionality could actively facilitate ester methanolysis rather than merely serve as a structural linker. Network‐level comparisons, model‐reaction kinetics, and computational analyses provide evidence for a previously unrecognized ether‐assisted and amine‐environment‐dependent molecular promotion mechanism for catalyst‐free methanolysis. This mechanistic understanding provides a design principle for regulating thermoset depolymerization through the local molecular environment surrounding cleavable ester bonds. The resulting networks combine tunable material properties with mild methanolysis, enabling the recovery of ester building blocks and polyols, with the latter further upcycled into antibacterial quaternary ammonium materials. Thus, this work links molecular design, methanolysis mechanism, material performance, and value‐added reuse within a single polyphenol‐derived platform.

## 2 Results and Discussion

### 2.1 Synthesis and Methanolysis Study of PPHA‐Based Epoxy‐Amine Thermosets

The biobased functional building blocks were primarily prepared from bio‐renewable polyphenols and α‐hydroxy acid derivatives in one step. Hydroquinone and catechol were used here as representative model polyphenols to demonstrate the feasibility of the molecular design strategy. Such phenolic structures are commonly present in lignin‐derived aromatics, and both compounds can also be obtained from biomass‐derived phenolic intermediates through lignin valorization pathways [57, 58, 59]. Subsequent transformation afforded PPHA‐derived epoxide monomers in overall yields of 36–55% on an approximately 10 g scale (see Scheme S1). Related epoxide monomers prepared via one‐step esterification of esters with glycidol have also been reported previously [60, 61]. Epoxide **M6**, lacking the ether groups, was prepared as a control monomer to evaluate the role and utility of the special ether functionalities in **M1–M5**. All epoxides (**M1–M6**) were characterized by NMR spectroscopy and ESI‐MS (see Figures S1−S12). Subsequently, a series of epoxy resins were prepared by curing the epoxides with diamines (Figure 2a). The curing process involved a pre‐curing step at 90°C for 3 h followed by post‐curing at 140°C for another 3 h. Fourier‐transform infrared (FTIR) spectroscopy confirmed the complete consumption of the epoxide groups (see Figures S16‐20), while gel content values > 98% further verified the successful formation of highly cross‐linked networks (see Table S1).

**FIGURE 2:**
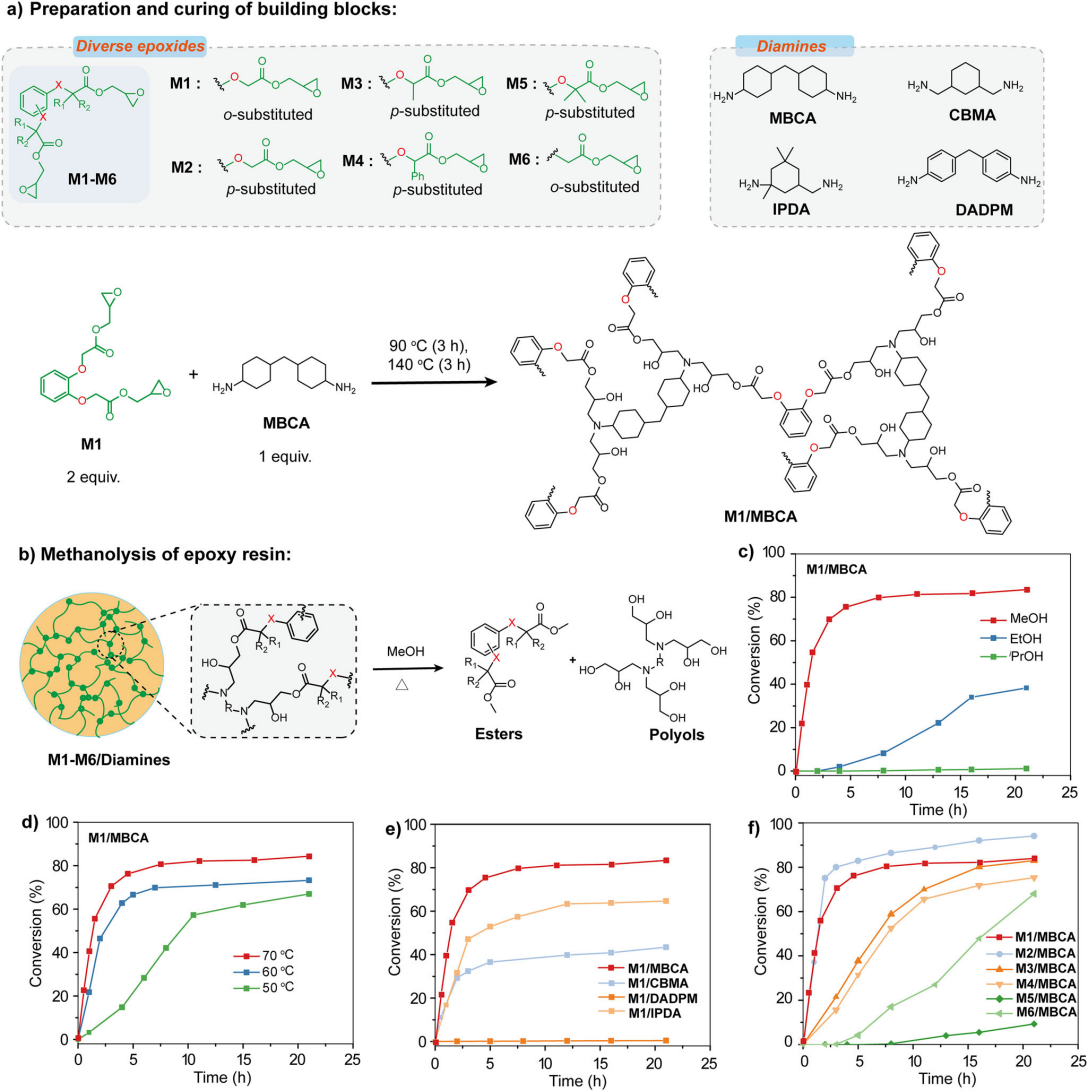
(a) Preparation and subsequent curing of building blocks: diverse epoxide monomers **M1‐M6** and selected diamines. (b) Schematic illustration of the methanolysis of epoxy resins with different formulations. (c) Kinetic plots of depolymerization of **M1/MBCA** with different alcohols at 70°C, monitored by ^1^H NMR conversion. (d) Kinetic plots of methanolysis of **M1/MBCA** at 50°C, 60°C, 70°C, monitored by ^1^H NMR conversion. (e) Kinetic plots of methanolysis of **M1/(MBCA**, **CBMA**, **DADPM**, **IPDA)** at 70°C, monitored by ^1^H NMR conversion. (f) Kinetic plots of methanolysis of **M1‐M6/MBCA** at 70°C, monitored by ^1^H NMR conversion.

To study the structure–depolymerization relationships of the resulting epoxy resins (Figures 2b and S21‐S40), different conditions such as temperature, solvent, and composition were investigated. **M1/MBCA** was selected as a representative epoxy resin, and its depolymerization was investigated in different alcohols at ∼70°C (Figure 2c). Surprisingly, methanol enabled rapid depolymerization of the epoxy resin, reaching ∼60% conversion within 2 h and ∼84% after 21 h to the corresponding methyl ester. In contrast, ethanol resulted in much lower efficiency, with conversion increasing gradually to only ∼40% after 21 h, while isopropanol showed negligible depolymerization even after prolonged reaction times. This effect likely arises from the small steric hindrance of methanol and its favorable polarity match with the epoxy resin [56]. Based on the solvent screening results, the effect of temperature on **M1/MBCA** depolymerization in methanol was further investigated (Figure 2d). A decrease in temperature led to a reduced depolymerization rate, affording ∼70% conversion at 60°C and ∼65% at 50°C after 21 h.

Under the optimized conditions (MeOH, 70°C), the effect of diamine structures on the methanolysis of **M1**‐based epoxy networks was examined using **CBMA**, **IPDA**, and **DADPM** (Figure 2e). **M1/CBMA** and **M1/IPDA** exhibited much slower depolymerization, reaching only ∼40% and ∼60% conversion after 21 h, respectively, whereas **M1/DADPM** showed almost no depolymerization. This pronounced difference is likely associated with the lower basicity of the aromatic amine environment and its less favorable local molecular arrangement for methanol activation. These results highlight the important roles of amine basicity and the surrounding molecular environment in governing depolymerization efficiency [56].

To probe the structure–depolymerization relationship, epoxy resins based on structurally distinct epoxy monomers (**M1–M6**) and cured with the same diamine **MBCA** were evaluated under identical depolymerization conditions (Figure 2f). In particular, **M1** was compared with **M6** to evaluate the influence of neighboring ether functionalities on network depolymerization. **M6/MBCA** showed relatively slow depolymerization, reaching ∼70% conversion after 21 h, whereas **M1/MBCA** underwent faster methanolysis under identical conditions. This comparison supports a beneficial role of the neighboring ether functionalities in promoting methanolysis, although the network‐level results alone do not establish the microscopic origin of this ether effect. The underlying molecular contributions were therefore further examined using small‐molecule kinetic studies and computational analysis. Subsequently, the influence of substitution position (*o*‐ vs *p*‐) was systematically investigated. The *p*‐substituted monomer **M2** cured with **MBCA** showed similar depolymerization behavior but reached a higher conversion of ∼90% within 12 h. This difference may be associated with the distinct substitution patterns. One possible explanation is that intramolecular cyclization may be more favorable for the *o*‐substituted M1, whereas the *p*‐substituted M2 may provide a more accessible network environment for methanolysis. Based on this, a series of *p*‐substituted **M3‐M5** bearing significant steric hindrance in the vicinity of the ester bonds were designed to elucidate the steric effects on depolymerization efficiency. A clear steric‐hindrance‐dependent trend in depolymerization behavior was observed, with the depolymerization rate decreasing progressively from **M2** to **M5**. Specifically, Me‐substituted **M3/MBCA** reached ∼83% conversion after 21 h, while Ph‐substituted **M4/MBCA** showed a further decrease to ∼75%. Both **M3/MBCA** and **M4/MBCA** attained higher conversions by prolonging the depolymerization time, approaching 90% and 86% after 46 h, respectively. In contrast, the di‐methyl‐substituted **M5/MBCA** consistently exhibited the lowest depolymerization efficiency. Consistent trends were also observed in hydrolytic stability tests, where increasing steric hindrance markedly enhanced resistance to acidic and basic aqueous conditions. In particular, the **M5/MBCA** network remained intact for at least 7 days, whereas **M1** and **M2** underwent rapid hydrolysis within 3 days (Figure S48). These observations are attributed to increased activation barriers at the ester bonds imposed by steric hindrance, which limits nucleophilic access and slows reaction kinetics, in accordance with trends described by Taft steric constants (Es) [62]. Collectively, these results demonstrate that rational redesign of polyphenol‐derived building blocks enables systematic and predictable control over depolymerization behavior, highlighting the generality of this molecular design strategy.

### 2.2 Investigation of the Methanolysis Mechanism

To further clarify the role of the ether linkage in methanolysis, two small‐molecule model compounds were designed, and their methanolysis was investigated in the presence of a representative tertiary amine model that mimics the local network environment (Figure 3a). **Model 1** was used as an ether‐free control, whereas **Model 2** contains an ether moiety adjacent to the aromatic ester structure. Temperature‐dependent kinetic studies showed that methanolysis was accelerated at elevated temperatures for both model compounds, as reflected by the increased apparent rate constants from 50 to 70°C (Figure 3b,c). These results indicated that temperature plays an important role in accelerating methanolysis, while the structural difference between the two models significantly affects the reaction kinetics. Arrhenius analysis based on the temperature‐dependent rate constants gave apparent activation energies of 48.2 and 38.2 kJ mol^−1^ for **Model 1** and **Model 2**, respectively (Figure 3d). The ether‐containing **Model 2** exhibited a lower apparent activation energy than the ether‐free **Model 1**, providing kinetic evidence that the neighboring ether moiety facilitates ester methanolysis by lowering the apparent kinetic barrier. However, the kinetic results alone do not establish the microscopic origin of this ether effect. Notably, the influence of the amine environment was further examined by replacing the cyclohexylamine‐type aliphatic amine in **Model 2** with an aniline‐type aromatic amine. Under otherwise identical conditions, the aromatic amine‐containing model showed only trace methanolysis conversion (Figure S47). In sharp contrast, the cyclohexylamine‐type aliphatic amine enabled nearly quantitative conversion within 2 h. These results suggest that efficient methanolysis requires the cooperative influence of the neighboring ether functionality and a favorable aliphatic amine environment. This observation is consistent with the network depolymerization results, where the aromatic diamine‐based **M1/DADPM** showed negligible depolymerization, whereas aliphatic amine‐based networks underwent much more efficient methanolysis.

**FIGURE 3:**
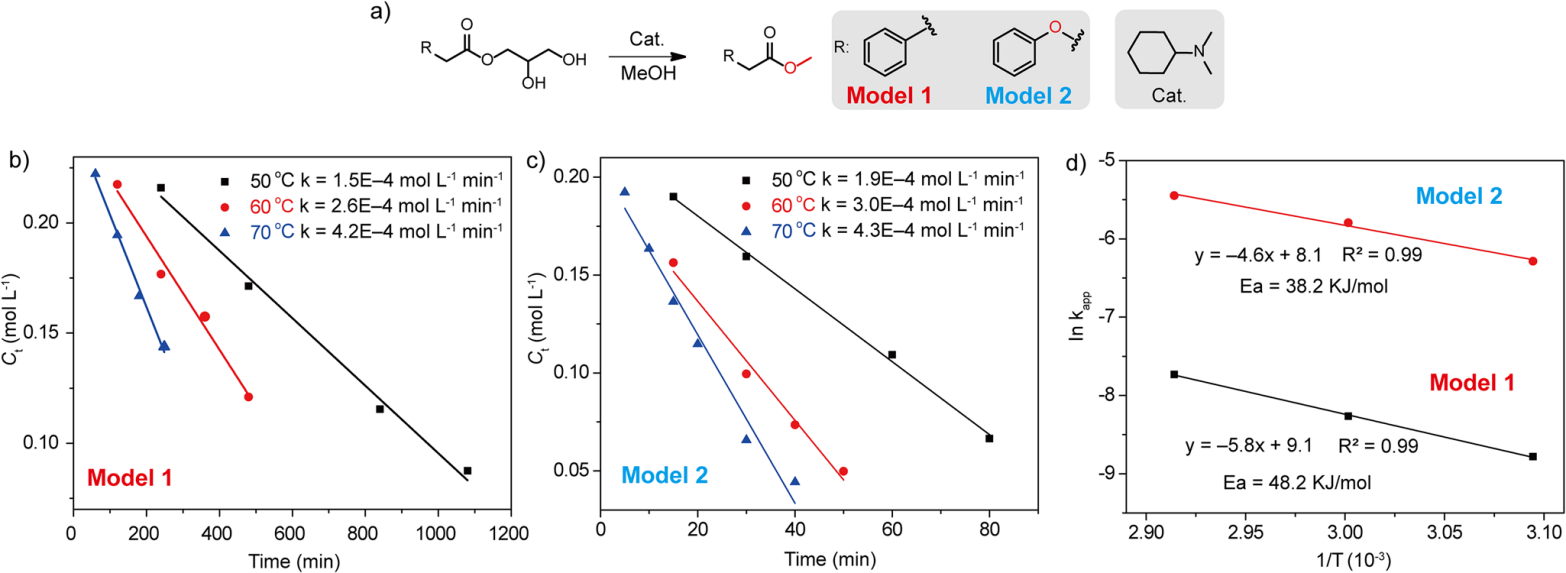
Mechanistic study of methanolysis. (a) Small‐molecule model compounds and amine model used to mimic the local methanolysis environment. (b, c) Temperature‐dependent kinetic fitting of methanolysis for **Model 1** and **Model 2**, respectively. (d) Arrhenius analysis of **Model 1** and **Model 2** methanolysis.

Density functional theory (DFT) calculations and Natural Population Analysis (NPA) further showed that the ether‐containing models did not exhibit increased carbonyl electrophilicity relative to their ether‐free analogues (see computational chapter in Supporting Information). Meanwhile, the methanol oxygen carried a more negative natural charge in all molecular complexes, although the extent of this polarization depended on the stereochemical configuration and the resulting local molecular arrangement. In the RR configuration, the methanol oxygen was more negatively charged in the ether‐containing model than in the ether‐free analogue (−0.785 versus −0.778), whereas the opposite trend was observed in the RS configuration (−0.759 versus −0.780). These results suggest that the ether effect is unlikely to originate from a simple inductive electronic effect, but may instead indicate a local‐structure‐dependent contribution of the neighboring ether functionality.

Taken together, efficient methanolysis in **PPHA**‐derived epoxy networks likely arises from the cooperative influence of a favorable aliphatic amine environment and configuration‐dependent modulation of the local interaction environment by the neighboring ether functionality. The increased conformational flexibility and accessibility of the ether‐containing structure may facilitate hydrogen‐bonding arrangements that modulate methanol polarization and positioning around the ester bond, thereby lowering the apparent kinetic barrier for catalyst‐free methanolysis. These findings support a previously unrecognized molecular promotion mechanism in which the neighboring ether functionality and the aliphatic amine environment cooperatively regulate the local reaction environment to facilitate ester methanolysis.

### 2.3 Thermal and Mechanical Properties

To further elucidate the substitution effect on the epoxy resin properties, thermal gravimetric analysis (TGA) and differential scanning calorimetry (DSC) were employed to investigate the thermal properties of the obtained polyphenol‐based **M1‐M5/MBCA**. They showed *T*_d_ (decomposition temperature, defined by the temperature of 5% weight loss) of 250°C–290°C (Figure 4a), displaying notable thermal stability. Their DSC curves (Figure 4b) revealed that these epoxy resins exhibited tunable thermal properties with glass‐transition temperatures (*T*_g_) in a range of 66°C–105°C. The lower *T*_g_ values compared with the DGEBA/MBCA reference network (153°C) are likely associated with the more flexible ester/ether‐containing **PPHA**‐derived structures, which reduce backbone rigidity and increase segmental mobility in the cured networks. The mechanical performance of these thermosets was evaluated by tensile testing (Figures 4c, S51‐54, and Tables S4‐8). **M1/MBCA** and **M4/MBCA** yielded defective specimens (brittleness/bubbles) and were excluded from tensile analysis. **M2/MBCA**, **M3/MBCA**, and **M5/MBCA** materials showed useful mechanical performance, with tensile strengths of 62–78 MPa, Young's moduli of 0.7–1.2 GPa, strains above 10%, and toughness values of 5.4–6.6 MJ m^−^^3^. The relatively ductile mechanical response may be associated with the incorporation of α‐hydroxy acid–derived flexible segments, which increase segmental mobility and facilitate energy dissipation during deformation [63, 64, 65]. Building on this, dynamic mechanical analysis (DMA) of these samples was also investigated (Figure 4d). Interestingly, the higher *T*_g_ values 95°C–114°C (defined by the peak maxima of tanδ) obtained from DMA compared to DSC arise from the fundamentally different evaluation criteria of the two techniques, with DMA probing mechanical relaxation processes rather than calorimetric transitions [66]. Furthermore, three of them showed a similar high storage modulus (>2.0 GPa). These results demonstrated that both thermal and mechanical properties can be tuned by varying the substitution position and substituent groups of the epoxy building blocks, enabling controlled property modulation. The cryo‐fractured cross‐sections of the epoxy networks were examined by scanning electron microscopy (SEM) (Figure 4e) to gain insight into the fracture morphology. **DGEBA/MBCA** showed a comparatively smooth fracture surface, suggesting a more brittle fracture mode. In contrast, the PPHA‐derived networks, especially **M2/MBCA**, **M3/MBCA**, and **M5/MBCA**, presented rougher and more irregular cross‐sectional morphologies. Such textured fracture surfaces indicated more tortuous crack propagation and improved energy dissipation during deformation. Figure 4f summarizes the overall material performance of the prepared epoxy resins using a radar chart. Compared with commercial **DGEBA/MBCA**, the incorporation of biobased adjustable building blocks in the epoxy system contributed to tunable recyclability, thermal, and mechanical properties.

**FIGURE 4:**
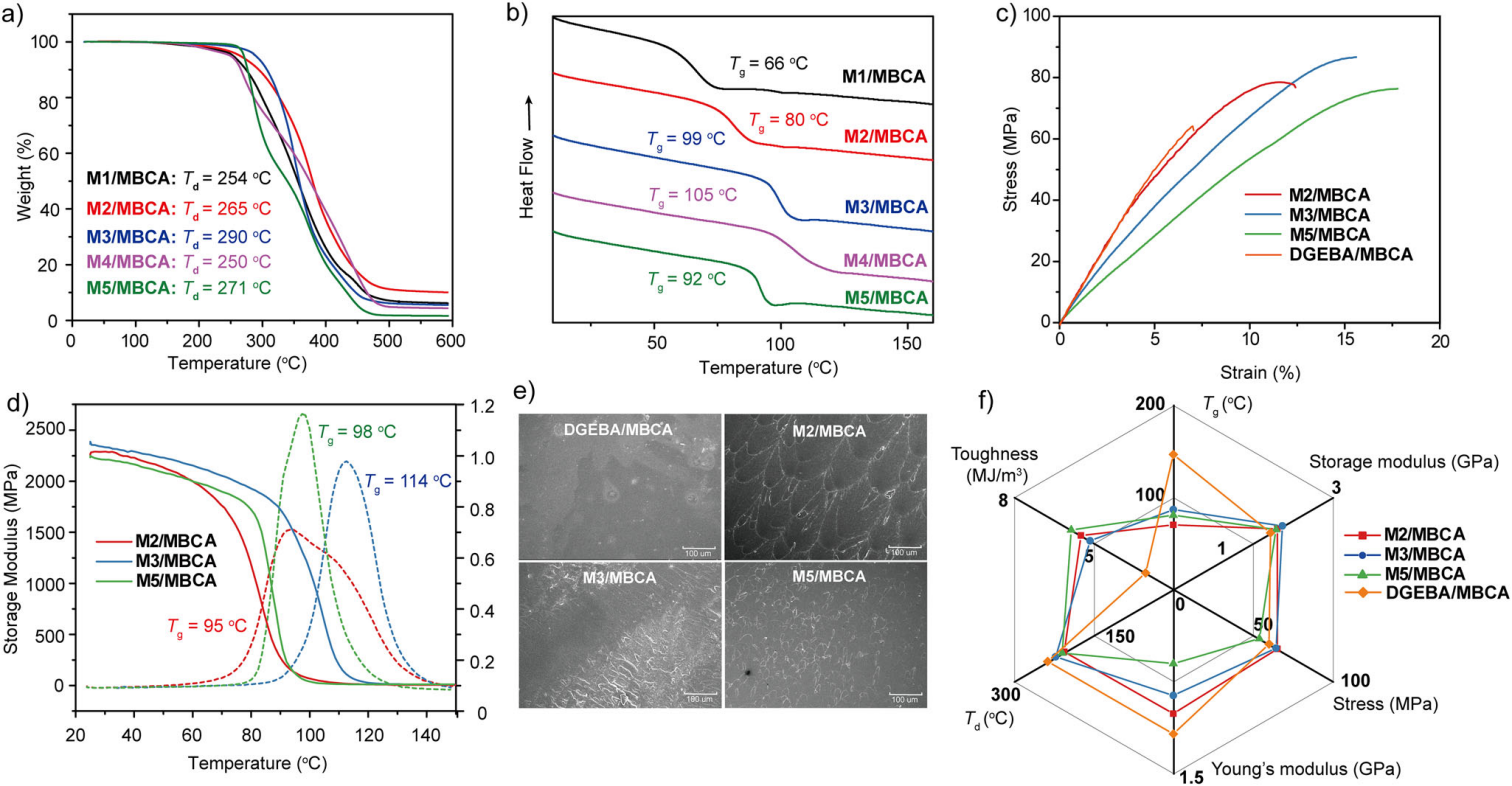
Material performance of epoxy resins. (a) TGA curves of **M1‐M5/MBCA**. (b) DSC curves of **M1‐M5/MBCA**. (c) Stress–strain curves of **M2/MBCA**, **M3/MBCA**, **M5/MBCA** and commercial **DGEBA/MBCA**. (d) Storage modulus and tan δ curves of **M2/MBCA**, **M3/MBCA**, and **M5/MBCA**. (e) SEM characterization of the cross‐sectional morphology of polymer samples (**M2/MBCA**, **M3/MBCA, M5/MBCA** and **DGEBA/MBCA**) after cryo‐fracturing in liquid nitrogen. (f) Radar chart comparing properties of **M2/MBCA**, **M3/MBCA** and **M5/MBCA** samples with **DGEBA/MBCA**.

### 2.4 Applications and Recycling of Carbon Fiber Composites

To demonstrate the applicability of the epoxy resin, carbon fiber–reinforced composites based on **M2/MBCA** were fabricated (Figure 5). The resulting composites were readily depolymerized by methanolysis within 48 h. SEM images of the pristine and recovered carbon fibers showed no observable resin residues on the fiber surfaces, indicating that the mild methanolysis conditions effectively removed the polymeric matrix without compromising the integrity of the carbon fibers. The degradation residues were separated using a DCM/petroleum ether mixture, affording recycled ester (1.35 g, 88%) and polyols—depolymerized tertiary amine alcohol (D‐TAA, 1.65 g). Their structures were confirmed by ^1^H NMR and ESI–MS analysis, with comparison to model polyols—synthesized tertiary amine alcohol (S‐TAA, Scheme S2 and Figures S55−S58) indicating predominantly secondary and tertiary amine functionalities. FTIR spectra of D‐TAA and S‐TAA showed weak ester and aromatic signals, together with weak absorptions that may be assigned to amide‐related vibrations, suggesting that a minor fraction of ester groups underwent aminolysis during curing (Figure S58). Despite this side reaction, the ester building blocks were recovered in 88% isolated yield, confirming that epoxy–amine addition remained the dominant curing pathway and that high methanolysis efficiency was largely retained. The incomplete recovery is therefore possibly attributed to the formation of a small amount of methanolysis‐resistant amide‐containing structures. Further optimization of the curing chemistry and methanolysis conditions may suppress this side reaction and improve the recovery yield.

**FIGURE 5:**
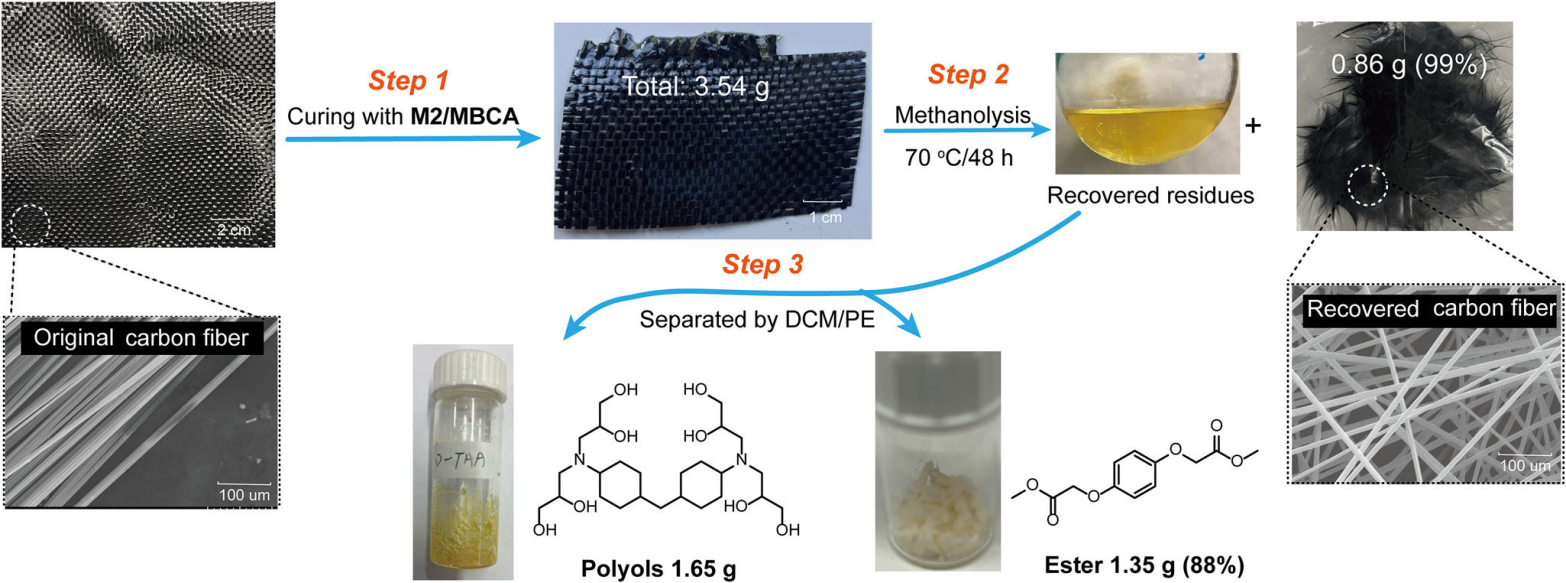
Synthesis and recyclability of **M2/MBCA**‐based carbon fiber composites. (Step 1) Curing condition: 2 equiv **M2**, 1 equiv **MBCA**, and carbon fibers, followed by curing at 90°C for 3 h and 140°C for 3 h. (Step 2) Depolymerization conditions: 3.54 g carbon fiber‐reinforced composites, 50 mL methanol, 70°C for 48 h. (Step 3) Separation of esters and polyols using DCM/petroleum ether, monitored by ^1^H NMR.

### 2.5 Upcycling and Antibacterial Application of the Recovered Polyols

Although methanolysis enables efficient depolymerization of epoxy–amine networks, the resulting polyols predominantly contain tertiary amine functionalities that are intrinsically difficult to convert back into the primary amines required for reconstructing epoxy–amine thermosets. Although Wu and co‐workers [56] demonstrated the possibility of recovering amine components from related epoxy systems, the process suffers from low recovery efficiency (22%) and requires harsh reaction conditions (acetic acid, 270°C), which limit its practical applicability. Therefore, rather than attempting direct regeneration of the original curing agents, we explored an alternative upcycling strategy for these polyols. Notably, the presence of tertiary amine motifs renders these polyols suitable precursors for quaternary ammonium materials [67, 68], enabling value‐added upcycling of the depolymerization products and maximizing the material circularity of the epoxy–amine system.

Accordingly, the depolymerization‐derived polyols bearing tertiary amine functionalities were converted into quaternary ammonium materials through quaternization with dibromoalkanes of different chain lengths (Figure 6a), while S‐TAA was synthesized as a reference compound. Successful quaternary ammonium material formation was confirmed by FTIR, ^1^H NMR, and zeta potential measurements, with charge increasing from −20 mV to above +30 mV after quaternization (Figures S59, S60, and Table S9). As shown in Figure 6b,c, pronounced antibacterial activity against MRSA was observed only for S‐TAA‐DBT and D‐TAA‐DBT, whereas the other materials with shorter or longer alkyl spacers (C_12_, C_18_), as well as the nonquaternized polyols, exhibited negligible antibacterial effects. In particular, S‐TAA‐DBT and D‐TAA‐DBT reduced MRSA viability by approximately 3–4 orders of magnitude at concentrations of 32 µg/mL, and by around 2–3 orders of magnitude at 16 µg/mL, demonstrating a clear concentration‐dependent antibacterial response (Figure 6c). These results indicated that an optimal hydrophilic–hydrophobic balance, governed by the alkyl chain length, is critical for effective antibacterial performance, affording minimum inhibitory concentrations (MICs) in the range of 16–32 µg/mL. Consistent with the planktonic antibacterial results, S‐TAA‐DBT and D‐TAA‐DBT effectively suppressed MRSA biofilm formation, whereas other materials showed only limited inhibition (Figure 6d). Importantly, all quaternized materials maintained high cytocompatibility toward A549 cells, with cell viability >80% even at concentrations up to 1000 µg/mL (Figure 6e), indicating a high selectivity balance between antibacterial efficacy (Figure 6c,d) and biocompatibility. Overall, this upcycling strategy demonstrated a viable route for the value‐added reutilization of recovered polyols, converting depolymerization products into functional antibacterial materials rather than merely low‐value products.

**FIGURE 6:**
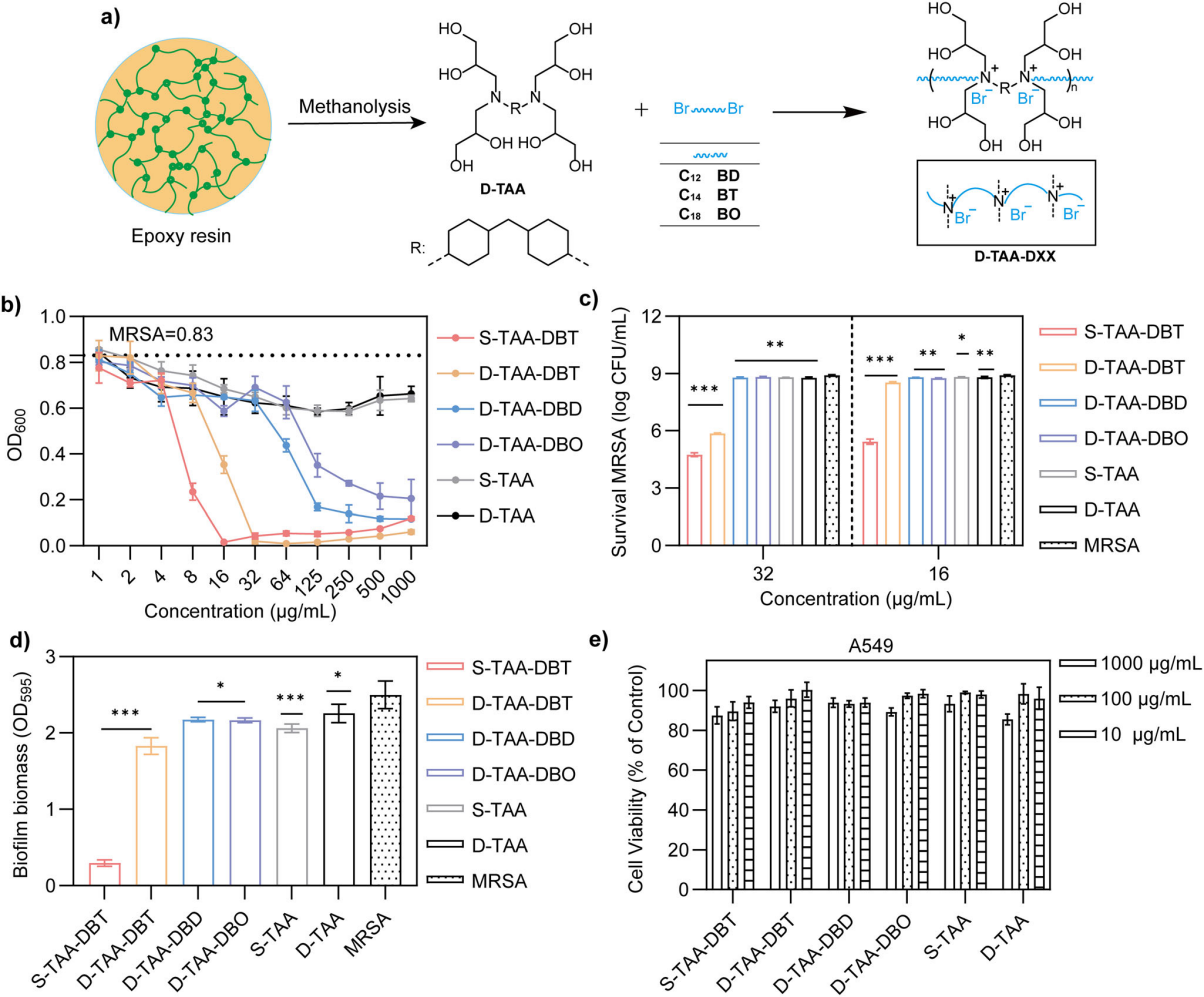
(a) Schematic illustration of the upcycling of recovered polyols into antibacterial quaternary ammonium materials. (b) Dose‐dependent growth inhibition of MRSA by different quaternary ammonium materials and control samples, evaluated by optical density at 600 nm (OD_600_) measurements after incubation. The dashed line indicates the OD_600_ value of MRSA without treatment. (c) Survival rate of MRSA after treatment with various materials at concentrations of 32 and 16 µg/mL. Statistical significance was analyzed in comparison with the MRSA control (*p* < 0.05, *p* < 0.01, *p* < 0.001). (d) Biofilm biomass of MRSA formed in the presence of different materials, quantified by OD_595_ measurements using crystal violet staining. Statistical significance was analyzed relative to the MRSA control (*p* < 0.05, *p* < 0.01, *p* < 0.001). e. In vitro cytocompatibility of the materials toward A549 cells after 24 h incubation at concentrations of 10, 100, and 1000 µg/mL, expressed as cell viability relative to the untreated control.

## 3 Conclusion

In summary, a facile synthetic route was utilized to prepare biobased functional building blocks (PPHA) with tunable substitution positions and groups through molecular design of biobased polyphenols, thereby enabling the construction of a diverse library of epoxide monomers with well‐defined and tailorable molecular architectures. These monomers (**M1–M5**) were subsequently cured with a diamine (**MBCA**) to construct diverse biobased epoxy–amine thermosets. Most importantly, this study supports a previously unrecognized ether‐assisted molecular promotion mechanism for methanolysis in epoxy–amine thermosets. Model‐reaction kinetics demonstrated that the neighboring ether functionality lowers the apparent kinetic barrier, while comparative experiments and computational analyses indicated that efficient methanolysis arises from the effects of the ether‐containing local environment and a favorable aliphatic amine environment. This mechanistic understanding shifts the design of recyclable thermosets from the simple incorporation of cleavable ester bonds toward deliberate regulation of the local molecular environment surrounding these bonds. The resulting thermosets exhibited tunable thermal properties and useful mechanical performance, while systematic variation of the building‐block structures enabled simultaneous regulation of network properties and depolymerization behavior. The optimized **M2/MBCA** was successfully combined with carbon fibers to fabricate fiber‐reinforced composites. Upon mild methanolysis, the epoxy matrix was selectively depolymerized, enabling the recovery of intact carbon fibers together with reusable building blocks (88% isolated yield). Beyond material recovery, the depolymerization‐derived polyols were further upcycled into antibacterial quaternary ammonium materials, establishing a value‐added reuse pathway that broadens the material circularity of epoxy–amine thermosets. Overall, this work establishes a modular polyphenol‐derived platform that links molecular structure, ether‐assisted catalyst‐free methanolysis, material performance, and value‐added reuse.

## Author Contributions

Y.‐M.T. and R.H. conceived the project and directed the research. Y.J. conducted antibacterial tests. Y.‐M.T. designed and conducted experiments related to polymer synthesis and depolymerization. K.L. and Q.C. conducted mechanical and thermal characterization. A.J.A. performed the density functional theory (DFT) calculations. Y.‐M.T. and A.J.A. wrote the initial draft of the manuscript. R.H. provided critical revisions and feedback. All authors contributed to the final version of the manuscript.

## Conflicts of Interest

The authors declare no conflicts of interest.

## Supporting information



Experimental details and supporting data are available in the Supporting Information.**Supporting File**: anie74694‐sup‐0001‐SuppMat.docx.

## Data Availability

The data that support the findings of this study are available in the Supporting Information of this article.
