# Supplementary material for: Reusable Polyphenol Building Blocks Enable Recyclable Epoxy‐Amine Thermosets via Methanolysis
Source: Angew Chem Int Ed Engl. 2026 Sep 16;65(44):e3352626. doi: 10.1002/anie.3352626 (PMC13703777; doi:10.1002/anie.3352626)
Supplement: Supplementary file 1 [file ANIE-65-e3352626-s001.docx]

**Supplemental Information**

**Reusable Polyphenol Building Blocks Enable Recyclable Epoxy-Amine Thermosets via Methanolysis**

Yi-Min Tu,*^[a]^ Yuhang Jiang,^[a]^ Kun Li,^[b]^ Qing Cao,^[b]^ Yizhe Pan,^[a]^ Christoph Tzschucke,^[a]^ Andreas J. Achazi,*^[a][c]^ and Rainer Haag*^[a]^

[a]	Dr. Y.-M. Tu, Y. Jiang. Y. Pan, Prof. Dr. C. Tzschucke, Dr. A. Achazi, Prof. Dr. K. Ludwig, Prof. Dr. R. Haag
Institute of Chemistry and Biochemistry, Freie Universität Berlin,
Takustraße 3, 14195 Berlin, Germany

[b]	K. Li, Q. Cao.
National Engineering Laboratory of Eco Friendly Polymeric Materials (Sichuan), State Key Laboratory of Advanced Polymer Materials, College of Chemistry, Sichuan University
29 Wangjiang Rd, Chengdu, 610064, P. R. China

[c]	Dr. A. J. Achazi

IT and Data Services

Zuse Institute Berlin

Takustraße 7, 14195 Berlin, Germany

**Table of Contents**

[Materials and Methods	3](#_Toc216874342)

[Column chromatography	3](#_Toc216874343)

[Spectroscopic Characterizations	3](#_Toc216874344)

[Fourier transform infrared spectroscopy (FT-IR)	3](#_Toc216874345)

[Thermal Analysis	3](#_Toc216874346)

[Tensile Test	4](#_Toc216874347)

[Gel content (GC)	4](#_Toc216874348)

[General Preparations	6](#_Toc216874349)

[General monomer synthesis procedure	6](#_Toc216874350)

[Model monomer synthesis	7](#_Toc216874351)

[NMR spectra of the produced monomers	8](#_Toc216874352)

[General curing procedures	20](#_Toc216874353)

[FT-IR characterization	20](#_Toc216874354)

[GC of different epoxy resins	23](#_Toc216874355)

[Depolymerization of epoxy resin via methanolysis	24](#_Toc216874356)

[General methanolysis procedures	24](#_Toc216874357)

[Methanolysis of **M1/MBCA** under different temperatures	24](#_Toc216874358)

[Methanolysis of **M1/MBCA** with different solvents	25](#_Toc216874359)

[Methanolysis of **M1** with different amines	25](#_Toc216874360)

[Methanolysis of **M1-M6/MBCA**	26](#_Toc216874361)

[^1^H NMR spectra of methanolysis of different epoxy resin	27](#_Toc216874362)

[Methanolysis of **M1/MBCA** at different temperatures	27](#_Toc216874363)

[Methanolysis of **M1/MBCA** with different solvents	28](#_Toc216874364)

[Methanolysis of **M1** with different amines	29](#_Toc216874365)

[Methanolysis of **M1-M6/MBCA**	31](#_Toc216874366)

[Thermal Properties of Polymers	34](#_Toc216874367)

[Thermo Gravimetric Analysis	35](#_Toc216874368)

[Differential Scanning Calorimetry Characterizations	35](#_Toc216874369)

[Mechanical Property	36](#_Toc216874370)

[Summary of tensile test	36](#_Toc216874371)

[Preparation of quaternary ammonium materials	40](#_Toc216874372)

[Synthesis of of tertiary amine alcohol (S-TAA)	40](#_Toc216874373)

[Tertiary amine alcohol (D-TAA) from the depolymerization of epoxy resin	40](#_Toc216874374)

[Synthesis and characterization of quaternary ammonium materials	44](#_Toc216874375)

[Characterization of quaternary ammonium materials	44](#_Toc216874376)

[References	46](#_Toc216874377)

# Materials and Methods

All the reagents and solvents were purchased from commercial suppliers and used without further purification.

## Column chromatography

Medium-pressure liquid chromatography (MPLC) was performed with a TELEDYNE ISCO Combi-Flash Rf200 using prepacked silica columns and cartridges from TELDYNE. UV response was monitored at 254 nm and 280 nm. As eluents, cyclohexane (99.5+% quality) and EtOAc (HPLC grade) were used.

## Spectroscopic Characterizations

NMR spectra were recorded on a Bruker AV II-400 MHz spectrometer. Chemical shifts for all spectra were referenced to internal solvent resonances and were reported as parts per million relate to SiMe_4_. NMR spectroscopy: ^1^H NMR and ^13^C NMR spectral data were recorded on JEOL (ECX 400) spectrometers in the reported deuterated solvents. The chemical shifts (δ) are listed in parts per million (ppm) and are reported relative to the corresponding residual non-deuterated solvent signal (CDCl_3_: δH = 7.26 ppm, δC = 77.2 ppm; CD_3_OD: δH = 3.31 ppm, δC = 49.00 ppm). Integrals are in accordance with assignments; coupling 1 constant (J) are given in Hz. Multiplicity is indicated as follows: s (singlet), d (doublet), t (triplet), q (quartet), p (pentet), br (broad), and combinations thereof.

## Fourier transform infrared spectroscopy (FT-IR)

Epoxide monomers with different content carbonates and different epoxy resin were recorded on a Perkin Elmer from 4000 cm^-1^ to 450 cm^-1^.

## Thermal Analysis

Glass-transition temperature (*T*_g_) of purified and thoroughly dried polymer samples were measured by differential scanning calorimetry (DSC) on DSC25, TA Instrument. *T*_g_ values were obtained from a second scan after the thermal history was removed from the first scan. The second heating rate was 10 °C/min and cooling rate was 10 °C/min. Decomposition temperature (*T*_d_) and maximum rate decomposition temperatures (*T*_max_) of purified and thoroughly dried polymer samples were measured by thermogravimetric analyzer (TGA) on an TGA55, TA Instrument. Polymer samples were heated from ambient temperatures to 600 °C at a heating rate of 10 °C/min. Values of *T*_max_ were obtained from derivative (wt%/°C) vs. temperature (°C) plots and defined by the peak values, while *T*_d_ values were obtained from wt% vs. temperature (°C) plots and defined by the temperature of 5% weight loss. Storage modulus (E′, MPa), loss modulus (E″, MPa) and tan δ (E″/E′) measurements for samples were performed on a DMA 850 equipment (TA Instruments) with an 18 N load cell in tension film mode on rectangular specimens at a heating rate of 3 °C per min.

## Tensile Test

Tensile stress/strain testing was performed by an Instron 34SC-1 universal testing system. Samples were stretched at a strain rate of 10 mm/min at ambient temperature until break. The measurements were performed 4-6 times for each test, and pick up 3-5 times similar data, and the average values with standard error were calculated.

## Gel content (GC)

Three samples from the same material, of around 100 mg each, were separately immersed in THF for 24 h. Then these samples were dried in a ventilated oven at 80 °C for 24 h. The gel content (GC) was calculated using the equation below, where m_2_ is the mass of the dried material and m_1_ is the initial mass. The reported gel content is the average value of the three samples. The average GC content was calculated according to the following formula **S1**.

$GC=\frac{m_{2}}{m_{1}}\times100\%$ **S1**

## Zetapotential

Zetapotential were measured using a Malvern Zetasizer Ultra (Malvern Instruments Limited, U.K.) at a constant temperature of 25 °C PBS buffer solution at a fixed concentration of 1 mg/mL.

## Microdilution Assay

The bacteria strains MRSA (DMSZ No. 13661) were streaked out on an LB agar‑plate and incubated over night at 37 °C. The next day one colony was picked and diluted in 5.00 mL LB‑medium. After incubation over night at 37 °C and 250 rpm the bacteria solution was transferred to a 5.00 ml falcon tube and centrifuged at room temperature for 8 min at 4500 rcf. The bacteria pellet was resuspended in fresh LB‑medium and the concentration of bacteria cells in solution was adjusted to c = 10^6^-10^7^ CFU/mL for both bacteria strains. Dilution rows of the cationic surfactants were prepared in LB‑medium on the 96‑well plate. Afterwards the bacteria solution was added to each well on the plate. As growth control bacteria without any treatment was added to the plate. As positive control 10% penicillin-streptomycin was added to the wells on the plate. A sterile control to exclude possible contaminations in the media was further included. The absorbance at 595 nm was measured before and after incubation overnight using an epoch2 plate‑reader. From comparing the absorbance before and after overnight incubation the bacterial growth was determined.

All measurements were performed with three technical and two or three biological repetitions. The bacterial growth was calculated by setting the bacterial growth without treatment to 100% and the positive control to 0%. All graphs were plotted using the GraphPad Prism 7 software.

## In vitro anti biofilm assay

For the biofilm formation inhibition assay, 190 μL of MRSA solution (10^6^ CFU/mL, LB medium) was introduced to each well in 96-well plate. Afterward, for each well, 10 μL different sample solutions (final concentration: 1 μg/mL C12-4Q, 1 μg/mL C18-4Q, 16 μg/mL vancomycin or PBS) were added and incubated for 72 hours under 37 ℃. The PBS group was set as the control group. Then, all mediums were removed, washed 3 times with PBS, and stained with 0.1 % crystal violet for 15 min at room temperature. Therefore, the biofilms were washed with RO water to remove the excess dye. After drying at room temperature, 200 μL of ethanol was used to redissolved the crystal violet in the bottom of each well. Finally, the optical density of each well at 600 nm was analyzed by microplate reader (Epoch 2).

For the biofilm eradication assay, we introduced 200 μL of MRSA solution (10^6^ CFU/mL, LB medium) to each well in 96-well plate and cultured them in 37 ℃ incubator for 72 hours to harvest mature biofilm. Then, for each well, all mediums were replaced with 100 μL different sample solutions (1 μg/mL C12-4Q, 1 μg/mL C18-4Q, 16 μg/mL vancomycin or PBS) for another 24 hours culture and afterward, removed all solutions and test for biofilm biomass using the same method as above.

Then, we tested biofilm viability. Briefly, we used the same methods to get mature biofilms with different treatments. Then, 100 μL WST work solution was added to each well and cultured for 3 hours in a 37 ℃ incubator. The absorbance at 455 nm was tested by a plate reader (Epoch 2). For live/dead staining, the procedure provided by the manufacturer (L13152, Thermofisher) was followed, and fluorescence images were obtained by confocal microscopy (Leica).

## Statistical analysis

All data were expressed as mean ± SD from at least triplicated experiments. Differences between the two groups were analyzed by a One-way ANOVA. P < 0.1 were considered statistically significant.

# General Preparations

## General monomer synthesis procedure

Scheme S1. Literature procedure^[1, 2]^ was modified for the synthesis of **M1-M5**.

Synthesis of **a**

To a stirred suspension of anhydrous K_2_CO_3_ (300 mmol, 3 equiv.) in dimethyl formamide (150 mL) was added diphenol (100 mmol, 1 equiv.) and substituted methyl (ethyl) acetate **1-5** (240−300 mmol, 2.4-3 equiv.). The mixture was stirred under reflux until a TLC analysis showed that the starting material was completely consumed. The reaction mixture was cooled down to RT, then filtrated and concentrated under reduced pressure to afford the crude product **a1-a5**. The crude product was directly used in the next step.

Synthesis of **b**

To the solution of crude product (**a**) was added with NaOH (300 mmol, 3 equiv.) and H_2_O (200 mL). The reaction mixture was stirred at RT for 3 h until it formed as a homogenous solution. Hydrochloric acid (12 N) was then added to adjust the solution to pH ≈ 1, and cooled to −10 ℃. The precipitate was filtered and washed with cold water to get the aimed products. The crude product **b** was dried under vacuum, and then directly used in the next step without further purification.

Synthesis of **c**

To the solution of crude product (**b**) was added with thionyl chloride (SOCl_2_) (4 equiv.) and DMF (5 drops). The reaction mixture was refluxed for overnight. After that, extra SOCl_2_ was removed under vacuum. The crude product **c** was directly used in the next step without further purification.

The synthesis of **M1-M5** (Method 1)

Crude carboxylic acid **b** (1 equiv.) was dissolved in 50 mL of anhydrous DCM to prepared the acyl chloride solution. Then it was slowly added to a cold DCM (150 mL) solution of triethylamine (Et_3_N) (3 equiv.) and glycidol (2.5 equiv.). After the addition, the solution was allowed to stir at −5 ℃ for 0.5 h − 1 h. After that, the solution was warmed up to RT and continued to stir at RT for 6 − 10 h. The organic solution was washed with water and saturated brine, dried over anhydrous Na_2_SO_4_, and concentrated under reduced pressure. The crude products were purified by Combiflash to afford **M1-M5**.

The synthesis of **M1-M5** (Method 2)

To a solution of crude carboxylic acid **b** (1 equiv.) in 150 mL of anhydrous DCM was added catalytic amount 4-Dimethylaminopyridine (DMAP) and glycidol (2.5 equiv.). The resulting mixture was cooled with ice wtaer, and 1-Ethyl-3-(3-dimethylaminopropyl)carbodiimide (EDCI) (2.4 equiv.) was added to the solution. After the addition, the solution was warmed up to RT and continued to stir at RT for 3 h-4 h. ^1^H NMR was used to monitor the reaction until no medium product existed in the solution. The organic solution was washed with water and saturated sodium bicarbonate, dried over anhydrous Na_2_SO_4_, and concentrated under reduced pressure. The crude products were purified by Combiflash to afford **M1-M5**.

## Model monomer synthesis

Synthesis of **M6**

To a solution of 1,2-Phenylenediacetic acid (1 equiv.) in 150 mL of anhydrous DCM was added catalytic amount DMAP and glycidol (2.5 equiv.). The resulting mixture was cooled with ice wtaer, and EDCI (2.4 equiv.) was added to the solution. After the addition, the solution was warmed up to RT and continued to stir at RT for 3 h−4 h. ^1^H NMR was used to monitor the reaction until no medium product existed in the solution. The organic solution was washed with water and saturated sodium bicarbonate, dried over anhydrous Na_2_SO_4_, and concentrated under reduced pressure. The crude products were purified by Combiflash to afford **M6**.

## Model molecular synthesis

**Model 1**:

*Step 1 and 2*: To a solution of crude carboxylic acid (1 equiv.) in 150 mL of anhydrous DCM was added catalytic amount DMAP and solketal (1.2 equiv.). The resulting mixture was cooled with ice wtaer, and EDCI (1.2 equiv.) was added to the solution. After the addition, the solution was warmed up to RT and continued to stir at RT for 3 h-4 h. ^1^H NMR was used to monitor the reaction until no medium product existed in the solution. The organic solution was washed with water and saturated sodium bicarbonate, dried over anhydrous Na_2_SO_4_, and concentrated under reduced pressure.

The crude product obtained from Step 3 was dissolved in THF to afford a homogeneous solution. To this solution, 1 N aqueous HCl solution 100 mL was added slowly at room temperature. The reaction mixture was stirred at room temperature until TLC analysis indicated complete consumption of the starting material. After completion, the mixture was transferred to a separatory funnel and extracted with ethyl acetate three times. The combined organic layers were dried over anhydrous Na₂SO₄, filtered, and concentrated under reduced pressure. The crude residue was purified by CombiFlash chromatography to afford the desired product (10 g, Yield: 48%).

**Model 2:**

*Step 1 and 2*: To a stirred suspension of anhydrous K_2_CO_3_ (150 mmol, 1.5 equiv.) in acetonitrile (150 mL) was added phenol (100 mmol, 1 equiv.) and ethyl bromoacetate (120 mmol, 1.2 equiv.). The mixture was stirred under reflux until a TLC analysis showed that the starting material was completely consumed. The reaction mixture was cooled down to RT, then filtrated and concentrated under reduced pressure to afford the crude product. The crude product was directly used in the next step.

To the crude product (**a**) was added with NaOH (150 mmol, 1.5 equiv.), H_2_O (100 mL) and MeOH (50 mL). The reaction mixture was stirred at RT for overnight. Hydrochloric acid (12 N) was then added to adjust the solution to pH ≈ 1, and cooled to −10 ℃. The precipitate was filtered and washed with cold water to get the aimed products. The crude product was dried under vacuum, and then directly used in the next step without further purification.

*Step 3 and 4*

To a solution of crude carboxylic acid (1 equiv.) in 150 mL of anhydrous DCM was added catalytic amount DMAP and solketal (1.2 equiv.). The resulting mixture was cooled with ice wtaer, and EDCI (1.2 equiv.) was added to the solution. After the addition, the solution was warmed up to RT and continued to stir at RT for 3 h − 4 h. ^1^H NMR was used to monitor the reaction until no medium product existed in the solution. The organic solution was washed with water and saturated sodium bicarbonate, dried over anhydrous Na_2_SO_4_, and concentrated under reduced pressure.

The crude product obtained from Step 3 was dissolved in THF to afford a homogeneous solution. To this solution, 1 N aqueous HCl solution 100 mL was added slowly at room temperature. The reaction mixture was stirred at room temperature until TLC analysis indicated complete consumption of the starting material. After completion, the mixture was transferred to a separatory funnel and extracted with ethyl acetate three times. The combined organic layers were dried over anhydrous Na₂SO₄, filtered, and concentrated under reduced pressure. The crude residue was purified by CombiFlash chromatography to afford the desired product (8 g, Yield: 36%).

## NMR spectra of the produced monomers

#### Characterizations of **M1**

Monomer Yield: 9.9 g; Total yield 56%; ^1^H NMR (400 MHz, CDCl_3_) δ 6.98 – 6.88 (m, 4H), 4.77 (s, 4H), 4.53 (dd, *J* = 12.2, 3.0 Hz, 2H), 4.02 (dd, *J* = 12.2, 6.3 Hz, 2H), 3.26 – 3.19 (m, 2H), 2.85 – 2.78 (m, 2H), 2.66 – 2.58 (m, 2H). ^13^C NMR (101 MHz, CDCl_3_) δ 168.76, 148.00, 122.81, 115.81, 66.54, 65.55, 49.07, 44.61. ESI-MS: calculated for C_16_H_18_O_8_Na [M + Na]^+^ 361.0894, found 361.0929.

Figure S1. ^1^H NMR (CDCl_3_, 25 ℃) spectrum of **M1**.

Figure S2. ^13^C NMR (CDCl_3_, 25 ℃) spectrum of **M1**.

#### Characterizations of **M2**

Monomer Yield: 10.8 g; 55%; ^1^H NMR (400 MHz, CDCl_3_) δ 6.86 (s, 4H), 4.62 (s, 4H), 4.55 (dd, *J* = 12.2, 3.0 Hz, 2H), 4.01 (dd, *J* = 12.2, 6.4 Hz, 2H), 3.26 – 3.18 (m, 2H), 2.87 – 2.82 (m, 2H), 2.66 – 2.60 (m, 2H). ^13^C NMR (101 MHz, CDCl_3_) δ 168.80, 152.76, 115.92, 65.92, 65.58, 49.03, 44.58. ESI-MS: calculated for C_16_H_18_O_8_Na [M + Na]^+^ 361.0894, found 361.0905.

Figure S3. ^1^H NMR (CDCl_3_, 25 ℃) spectrum of **M2**.

Figure S4. ^13^C NMR (CDCl_3_, 25 ℃) spectrum of **M2**.

#### Characterizations of **M3**

Monomer Yield: 9.1 g; 51%; ^1^H NMR (400 MHz, CDCl_3_) δ 6.81 (s, 4H), 4.71 (q, *J* = 6.8 Hz, 2H), 4.54 – 4.40 (m, 2H), 4.05 – 3.92 (m, 2H), 3.22 – 3.11 (m, 2H), 2.85 – 2.75 (m, 2H), 2.64 – 2.50 (m, 2H), 1.65 – 1.55 (m, 6H). ^13^C NMR (100 MHz, CDCl_3_) δ 172.04, 152.45, 116.62, 116.61, 116.60, 116.59, 73.39, 65.42, 65.41, 49.13, 49.09, 44.56, 18.67, 18.65. ESI-MS: calculated for C_18_H_22_O_8_Na [M + Na]^+^ 389.1207, found 389.1227.

Figure S5. ^1^H NMR (CDCl_3_, 25 ℃) spectrum of **M3**.

Figure S6. ^13^C NMR (CDCl_3_, 25 ℃) spectrum of **M3**.

#### Characterizations of **M4**

Monomer Yield: 9 g; 36%; ^1^H NMR (400 MHz, CDCl_3_) δ 7.62 – 7.51 (m, 4H), 7.45 – 7.31 (m, 6H), 6.87 (s, 4H), 5.58 (s, 2H), 4.51 – 4.38 (m, 2H), 4.05 – 3.94 (m, 2H), 3.18 – 3.03 (m, 2H), 2.77 – 2.70 (m, 2H), 2.54 – 2.46 (m, 2H). ^13^C NMR (100 MHz, CDCl_3_) δ 169.69, 169.68, 152.26, 152.25, 135.31, 135.29, 129.12, 129.10, 128.85, 128.83, 127.11, 116.90, 116.89, 116.86, 116.84, 79.33, 79.29, 65.72, 65.38, 49.07, 48.96, 44.40, 44.36. ESI-MS: calculated for C_28_H_26_O_8_Na [M + Na]^+^ 513.1520, found 513.1515.

Figure S7. ^1^H NMR (CDCl_3_, 25 ℃) spectrum of **M4**

Figure S8. ^13^C NMR (CDCl_3_, 25 ℃) spectrum of **M4**.

#### Characterizations of **M5**

Monomer Yield: 12 g; 52%; ^1^H NMR (400 MHz, CDCl_3_) δ 6.77 (s, 4H), 4.46 (dd, *J* = 12.2, 3.2 Hz, 2H), 4.05 (dd, *J* = 12.2, 6.0 Hz, 2H), 3.21 – 3.13 (m, 2H), 2.84 – 2.78 (m, 2H), 2.64 – 2.57 (m, 2H), 1.56 (s, 12H). ^13^C NMR (100 MHz, CDCl_3_) δ 174.04, 150.62, 120.93, 79.70, 65.59, 49.17, 44.66, 25.47. ESI-MS: calculated for C_20_H_26_O_8_Na [M + Na]^+^ 417.1520, found 417.1545.

Figure S9. ^1^H NMR (CDCl_3_, 25 ℃) spectrum of **M5**

Figure S10. ^13^C NMR (CDCl_3_, 25 ℃) spectrum of **M5**.

#### Characterizations of **M6**

Monomer Yield: 10 g; 68%; ^1^H NMR (400 MHz, CDCl_3_) δ 7.26 (s, 4H), 4.43 (dd, *J* = 12.3, 2.9 Hz, 2H), 3.91 (dd, *J* = 12.3, 6.3 Hz, 2H), 3.75 (s, 4H), 3.23 – 3.13 (m, 2H), 2.84 – 2.77 (m, 2H), 2.63 – 2.53 (m, 2H). ^13^C NMR (100 MHz, CDCl_3_) δ 170.74, 132.80, 130.79, 127.64, 65.14, 65.13, 49.06, 44.32, 38.44. ESI-MS: calculated for C_16_H_18_O_6_Na [M + Na]^+^ 329.0996, found 329.1015.

Figure S11. ^1^H NMR (CDCl_3_, 25 ℃) spectrum of **M6**

Figure S12. ^13^C NMR (CDCl_3_, 25 ℃) spectrum of **M6**.

#### Characterizations of **Model 1**

^1^H NMR (400 MHz, CDCl_3_) δ 7.36 – 7.18 (m, 5H), 4.14 (dd, *J* = 5.5, 4.0 Hz, 2H), 3.93 – 3.81 (m, 1H), 3.65 (s, 2H), 3.60 (dd, *J* = 11.6, 3.7 Hz, 1H), 3.49 (dd, *J* = 11.6, 6.0 Hz, 1H), 2.85 (s, 2H).

Figure S13. ^1^H NMR (CDCl_3_, 25 ℃) spectrum of **Model 1**.

#### Characterizations of **Model 2**

^1^H NMR (400 MHz, CDCl_3_) δ 7.34 – 7.26 (m, 2H), 7.04 – 6.95 (m, 1H), 6.93 – 6.83 (m, 2H), 4.66 (s, 2H), 4.33 – 4.16 (m, 2H), 3.91 (t, *J* = 4.3 Hz, 1H), 3.67 – 3.48 (m, 2H), 2.78 (s, 3H). ^13^C NMR (100 MHz, CDCl_3_) δ 169.62, 169.61, 157.67, 129.79, 129.77, 129.74, 122.05, 114.78, 114.77, 114.68, 114.30, 69.99, 66.00, 65.37, 65.26, 63.31.

Figure S14. ^1^H NMR (CDCl_3_, 25 ℃) spectrum of **Model 2**.

Figure S15. ^13^C NMR (CDCl_3_, 25 ℃) spectrum of **Model 2**.

## General curing procedures

Cured reagents:

In the typical procedures: A range of different epoxy resins were synthesized by using epoxides (E) and different diamines (DA) as a curing agent (E: DA = 2:1). After the diamines and epoxides were thoroughly mixed at 90 ℃ for 3 h, the mixture was then placed in PTFE mold and cured at 140 °C for 3 h.

## **FT-IR characterization**

Figure S16. FT-IR spectra of epoxide monomer (**M1**) and epoxy resin (**M1-MBCA**). The marked part indicates the disappearance of the epoxy group.

Figure S17. FT-IR spectra of epoxide monomer (**M2**) and epoxy resin (**M2-MBCA**). The marked part indicates the disappearance of the epoxy group.

Figure S18. FT-IR spectra of epoxide monomer (**M3**) and epoxy resin (**M3-MBCA**). The marked part indicates the disappearance of the epoxy group.

Figure S19. FT-IR spectra of epoxide monomer (**M4**) and epoxy resin (**M4-MBCA**). The marked part indicates the disappearance of the epoxy group.

Figure S20. FT-IR spectra of epoxide monomer (**M5**) and epoxy resin (**M5-MBCA**). The marked part indicates the disappearance of the epoxy group.

## GC of different epoxy resins

Table S1. Gel contents of different epoxy resins

| Entry | Samples | Three times of gel contents (%) | | | GC (%) |
| --- | --- | --- | --- | --- | --- |
|  |  | 1 | 2 | 3 |  |
| 1 | **M1-MBCA** | 98.9 | 99.6 | 98.6 | 99.0±0.4 |
| 2 | **M2-MBCA** | 98.5 | 97.9 | 98.1 | 98.2±0.2 |
| 3 | **M3-MBCA** | 99.8 | 99.6 | 99.7 | 99.7±0.1 |
| 4 | **M4-MBCA** | 98.4 | 98.0 | 97.6 | 98.0±0.3 |
| 5 | **M5-MBCA** | 99.6 | 99.5 | 97.2 | 98.8±1.1 |

# **Depolymerization of epoxy resin via** **methanolysis**

## General **methanolysis** procedures

In the typical procedures: 100 mg of epoxy resin and 20 mg of the internal standard (hexamethylbenzene) were weighed into a 10 mL sample vial. Then, 4 mL of methanol solution was added, and the mixture was heated to 70 °C. At predetermined time intervals, small aliquots were withdrawn, concentrated, and analyzed by NMR spectroscopy. The conversion of the target product was calculated using the internal standard.

Figure S 21. Schematic diagram of depolymerization of epoxy resin via methanolysis.

### Methanolysis of **M1/MBCA** under different temperatures

Figure S 22. Comparative methanolysis of **M1/MBCA** at different temperatures.

### Methanolysis of **M1/MBCA** with different solvents

Figure S 23. Comparative alcoholysis of **M1/MBCA** in different solvents at 70 ℃.

### Methanolysis of **M1** with different amines

Figure S 24. Comparative methanolysis of **M1** with **MBCA, CBMA, DADPM, IPDA** and **DACH** at 70 ℃.

### Methanolysis of **M1-M6/MBCA**

Figure S 25. Comparative methanolysis of **M1-M6** with **MBCA** at 70 ℃.

Figure S 26. Comparative methanolysis of **M3,M4** with **MBCA** at 70 ℃.

## ^1^H NMR spectra of **methanolysis of different epoxy resin**

### Methanolysis of **M1/MBCA** at different temperatures

Figure S27. ^1^H NMR spectra (400 MHz, CDCl_3_) of methanolysis of **M1/MBCA** at 70 ℃ monitored over 21 h. The integration ratio of the resonance around 3.80 ppm and the resonance around 2.23 ppm (internal standard) was used to calculate the conversion of methanolysis.

Figure S28. ^1^H NMR spectra (400 MHz, CDCl_3_) of methanolysis of **M1/MBCA** at 60 ℃ monitored over 21 h. The integration ratio of the resonance around 3.80 ppm and the resonance around 2.23 ppm (internal standard) was used to calculate the conversion of methanolysis.

Figure S29. ^1^H NMR spectra (400 MHz, CDCl_3_) of methanolysis of **M1/MBCA** at 50 ℃ monitored over 21 h. The integration ratio of the resonance around 3.80 ppm and the resonance around 2.23 ppm (internal standard) was used to calculate the conversion of methanolysis.

### Methanolysis of **M1/MBCA** with different solvents

Figure S30. ^1^H NMR spectra (400 MHz, CDCl_3_) of alcoholysis (EtOH) of **M1/MBCA** at 70 ℃ monitored over 21 h. The integration ratio of the resonance around 4.73 ppm and the resonance around 2.23 ppm (internal standard) was used to calculate the conversion of methanolysis.

Figure S31. ^1^H NMR spectra (400 MHz, CDCl_3_) of alcoholysis (*^i^*PrOH) of **M1/MBCA** at 70 ℃ monitored over 21 h. The integration ratio of the resonance around 4.73 ppm and the resonance around 2.23 ppm (internal standard) was used to calculate the conversion of methanolysis.

### Methanolysis of **M1** with different amines

Figure S32. ^1^H NMR spectra (400 MHz, CDCl_3_) of methanolysis of **M1/CBMA** at 70 ℃ monitored over 21 h. The integration ratio of the resonance around 3.80 ppm and the resonance around 2.23 ppm (internal standard) was used to calculate the conversion of methanolysis.

Figure S33. ^1^H NMR spectra (400 MHz, CDCl_3_) of methanolysis of **M1/DACH** at 70 ℃ monitored over 21 h. The integration ratio of the resonance around 3.80 ppm and the resonance around 2.23 ppm (internal standard) was used to calculate the conversion of methanolysis.

Figure S34. ^1^H NMR spectra (400 MHz, CDCl_3_) of methanolysis of **M1/DADPM** at 70 ℃ monitored over 21 h. The integration ratio of the resonance around 3.80 ppm and the resonance around 2.23 ppm (internal standard) was used to calculate the conversion of methanolysis.

Figure S35. ^1^H NMR spectra (400 MHz, CDCl_3_) of methanolysis of **M1/IPDA** at 70 ℃ monitored over 21 h. The integration ratio of the resonance around 3.80 ppm and the resonance around 2.23 ppm (internal standard) was used to calculate the conversion of methanolysis.

### Methanolysis of **M1-M6/MBCA**

Figure S36. ^1^H NMR spectra (400 MHz, CDCl_3_) of methanolysis of **M2/MBCA** at 70 ℃ monitored over 21 h. The integration ratio of the resonance around 3.80 ppm and the resonance around 2.23 ppm (internal standard) was used to calculate the conversion of methanolysis.

Figure S37. ^1^H NMR spectra (400 MHz, CDCl_3_) of methanolysis of **M3/MBCA** at 70 ℃ monitored over 21 h. The integration ratio of the resonance around 3.80 ppm and the resonance around 2.23 ppm (internal standard) was used to calculate the conversion of methanolysis.

Figure S38. ^1^H NMR spectra (400 MHz, CDCl_3_) of methanolysis of **M4/MBCA** at 70 ℃ monitored over 21 h. The integration ratio of the resonance around 3.80 ppm and the resonance around 2.23 ppm (internal standard) was used to calculate the conversion of methanolysis.

Figure S39. ^1^H NMR spectra (400 MHz, CDCl_3_) of methanolysis of **M5/MBCA** at 70 ℃ monitored over 21 h. The integration ratio of the resonance around 3.80 ppm and the resonance around 2.23 ppm (internal standard) was used to calculate the conversion of methanolysis.

Figure S40. ^1^H NMR spectra (400 MHz, CDCl_3_) of methanolysis of **M6/MBCA** at 70 ℃ monitored over 21 h. The integration ratio of the resonance around 3.80 ppm and the resonance around 2.23 ppm (internal standard) was used to calculate the conversion of methanolysis.

# **Depolymerization of Model molecules via methanolysis**

## General **methanolysis** procedures

In the typical procedures: 1 mmol model molecules, 5 mmol% Cat. and 10 mg of the internal standard (hexamethylbenzene) were weighed into a 10 mL sample vial. Then, 4 mL of methanol solution was added, and the mixture was heated to 50, 60 and 70 °C. At predetermined time intervals, small aliquots were withdrawn, concentrated, and analyzed by NMR spectroscopy. The conversion of the target product was calculated using the internal standard.

### Methanolysis results of different models with catalyst

Table S2. Methanolysis results of **Model 1** with **Cat. 1** under different temperatures.

| Entry | Samples | Temperature (℃) | Time (h) | Conversion (%) |
| --- | --- | --- | --- | --- |
| 1 | **Model 1** | 50 | 4 | 13.6 |
|  |  |  | 8 | 31.5 |
|  |  |  | 14 | 53.8 |
|  |  |  | 18 | 65.0 |
| 2 |  | 60 | 2 | 13.0 |
|  |  |  | 4 | 29.3 |
|  |  |  | 6 | 37 |
|  |  |  | 8 | 51.6 |
| 3 |  | 70 | 1 | 11.1 |
|  |  |  | 2 | 22.2 |
|  |  |  | 3 | 33.3 |
|  |  |  | 4 | 42.5 |

Table S3. Methanolysis results of **Model 2** under different temperatures.

| Entry | Samples | Temperature (℃) | Time (min) | Conversion (%) |
| --- | --- | --- | --- | --- |
| 1^a^ | **Model 2** | 50 | 15 | 24.0 |
|  |  |  | 30 | 36.2 |
|  |  |  | 60 | 56.3 |
|  |  |  | 80 | 73.4 |
| 2^a^ |  | 60 | 15 | 37.5 |
|  |  |  | 30 | 60.2 |
|  |  |  | 40 | 70.6 |
|  |  |  | 50 | 80.1 |
| 3^a^ |  | 70 | 5 | 23.1 |
|  |  |  | 10 | 34.6 |
|  |  |  | 15 | 45.4 |
|  |  |  | 20 | 54.1 |
|  |  |  | 30 | 73.7 |
|  |  |  | 40 | 82.3 |
|  |  |  | 120 | >99 |
| 4^b^ |  | 70 | 120 | trace |

a: **Cat. 1**; b: **Cat. 2.**

## ^1^H NMR spectra of **methanolysis of different molecules**

### Methanolysis of **Model 1** at different temperatures

Figure S41. ^1^H NMR spectra (400 MHz, CDCl_3_) of methanolysis of **Model 1** at 50 ℃ monitored over 18 h. The integration ratio of the resonance around 4.10 − 4.30 ppm and the resonance around 2.23 ppm (internal standard) was used to calculate the conversion of methanolysis.

Figure S42. ^1^H NMR spectra (400 MHz, CDCl_3_) of methanolysis of **Model 1** at 60 ℃ monitored over 18 h. The integration ratio of the resonance around 4.10 − 4.30 ppm and the resonance around 2.23 ppm (internal standard) was used to calculate the conversion of methanolysis.

Figure S43. ^1^H NMR spectra (400 MHz, CDCl_3_) of methanolysis of **Model 1** at 70 ℃ monitored over 4 h. The integration ratio of the resonance around 4.10 − 4.30 ppm and the resonance around 2.23 ppm (internal standard) was used to calculate the conversion of methanolysis.

### Methanolysis of **Model 2** at different temperatures

Figure S44. ^1^H NMR spectra (400 MHz, CDCl_3_) of methanolysis of **Model 2** at 50 ℃ monitored over 80 min. The integration ratio of the resonance around 4.20 ppm and the resonance around 4.15 ppm was used to calculate the conversion of methanolysis.

Figure S45. ^1^H NMR spectra (400 MHz, CDCl_3_) of methanolysis of **Model 2** at 60 ℃ monitored over 50 min. The integration ratio of the resonance around 4.10 − 4.30 ppm and the resonance around 2.23 ppm (internal standard) was used to calculate the conversion of methanolysis.

Figure S46. ^1^H NMR spectra (400 MHz, CDCl_3_) of methanolysis of **Model 2** at 70 ℃ monitored over 40 min. The integration ratio of the resonance around 4.10 − 4.30 ppm and the resonance around 2.23 ppm (internal standard) was used to calculate the conversion of methanolysis.

**Cat. 2** 2h. Yield: trace

**Cat. 1** 2h. Yield: > 99%

Figure S47. ^1^H NMR spectra (400 MHz, CDCl_3_) of methanolysis of **Model 2** with **Cat. 1** and **Cat. 2** at 70 ℃ monitored over 2 h. The integration ratio of the resonance around 4.10 − 4.30 ppm and the resonance around 2.23 ppm (internal standard) was used to calculate the conversion of methanolysis.

# Acid-base stability test of epoxy resin

Cured epoxy resin specimens were immersed in aqueous acidic or basic solutions (1.0 M HCl or 1.0 M NaOH) at room temperature under static conditions. At selected time intervals (e.g., 1, 3, 5, and 7 days), photographs were taken directly in the solutions, and acid–base stability was qualitatively assessed based on changes in macroscopic appearance.

Figure S48. Acid–base stability of cured epoxy resins. Photographs of **M1–M5/MBCA** specimens with equal mass immersed in aqueous 1 M HCl (left) and 1 M NaOH (right) at room temperature under static conditions. Images were taken directly in the solutions at representative time intervals (from top to bottom). The apparent degradation of the epoxy resins gradually slows from **M1/MBCA** to **M5/MBCA**, as reflected by increasingly delayed macroscopic changes.

# Thermal Properties of Polymers

## Thermal Gravimetric Analysis

Figure S49. TGA curves of cured **M1-M5/MBCA.**

## Differential Scanning Calorimetry Characterizations

Figure S50. DSC curves (second heating scan) of cured **M1-M5/MBCA.**

# Mechanical Property

## Summary of tensile test

Table S4. Summary of mechanical properties of different epoxy resins.*^a^*

| Entry | Polymers | E/GPa | *σ*_b_/MPa | Elongation/% | Toughness/MJ m^-3^ |
| --- | --- | --- | --- | --- | --- |
| 1 | **M2-MBCA** | 1.2±0.1 | 77.8±2.4 | 11.0±1.1 | 5.50±0.90 |
| 2 | **M3-MBCA** | 1.0±0.1 | 74.1±6.6 | 11.9±2.1 | 5.41±1.66 |
| 3 | **M5-MBCA** | 0.7±0.1 | 62.1±3.2 | 15.7±1.5 | 6.63±1.06 |

*^a^*Condition: tested by uniaxial tensile tests. Strain rate of 10 mm/min, E: Tensile modulus, *σ*_b_: break strength.

#### Extension test of **M2-MBCA**

Figure S51. Stress-strain curve of **M2-MBCA** (Black curves: cured at 140 °C overnight; red curves: cured at 140 °C for 3 h).

Table S5. Summary of mechanical properties of **M2-MBCA***^a^*.

| Entry | E/GPa | *σ*_b_/MPa | Elongation/% | Toughness/MJ m^-3^ |
| --- | --- | --- | --- | --- |
| 1 | 1.19 | 72.6 | 13.0 | 5.06 |
| 2 | 1.17 | 74.4 | 10.6 | 6.47 |
| 3 | 1.14 | 78.5 | 12.5 | 6.38 |
| 4 | 1.17 | 79.9 | 10.7 | 4.08 |
| 5 | 1.14 | 80.4 | 11.7 | 5.00 |
| 6 | 1.29 | 75.6 | 9.3 | 5.51 |
| Average | 1.2 | 77.8 | 11.0 | 5.50 |
| Sdev | 0.1 | 2.4 | 1.1 | 0.90 |

*^a^*Condition: Tested by uniaxial tensile tests. Strain rate of 10 mm/min, E: Tensile modulus. *σ*_b_: break strength.

#### Extension test of **M3-MBCA**

Figure S52. Stress-strain curve of **M3-MBCA**.

Table S6. Summary of mechanical properties of **M3-MBCA***^a^*.

| Entry | E/GPa | *σ*_b_/MPa | Elongation/% | Toughness/MJ m^-3^ |
| --- | --- | --- | --- | --- |
| 1 | 1.07 | 66.7 | 10.2 | 4.32 |
| 2 | 0.97 | 67.3 | 11.4 | 4.85 |
| 3 | 0.83 | 73.4 | 13.4 | 5.98 |
| 4 | 1.07 | 81.0 | 9.2 | 3.82 |
| 5 | 0.84 | 82.2 | 15.1 | 8.10 |
| Average | 1.0 | 74.1 | 11.9 | 5.41 |
| Sdev | 0.1 | 6.6 | 2.1 | 1.66 |

*^a^*Condition: Tested by uniaxial tensile tests. Strain rate of 10 mm/min, E: Tensile modulus. *σ*_b_: break strength.

#### Extension test of **M5-MBCA**

Figure S53. Stress-strain curve of **M5-MBCA**.

Table S7. Summary of mechanical properties of **M5-MBCA***^a^*.

| Entry | E/GPa | *σ*_b_/MPa | Elongation/% | Toughness/MJ m^-3^ |
| --- | --- | --- | --- | --- |
| 1 | 0.59 | 61.9 | 16.3 | 6.60 |
| 2 | 0.70 | 57.7 | 14.6 | 5.53 |
| 3 | 0.60 | 62.0 | 17.8 | 8.07 |
| 4 | 0.84 | 66.8 | 13.9 | 6.30 |
| Average | 0.7 | 62.1 | 15.7 | 6.63 |
| Sdev | 0.1 | 3.2 | 1.5 | 1.06 |

*^a^*Condition: Tested by uniaxial tensile tests. Strain rate of 10 mm/min, E: Tensile modulus. *σ*_b_: break strength.

#### Extension test of **DGEBA-MBCA**

Figure S54. Stress-strain curve of **DGEBA-MBCA**.

Table S8. Summary of mechanical properties of **DGEBA-MBCA***^a^*.

| Entry | E/GPa | *σ*_b_/MPa | Elongation/% | Toughness/MJ m^-3^ |
| --- | --- | --- | --- | --- |
| 1 | 1.31 | 59.3 | 5.5 | 1.88 |
| 2 | 1.20 | 64.8 | 7.0 | 2.50 |
| 3 | 1.58 | 45.0 | 4 | 1.07 |
| Average | 1.36 | 56.4 | 5.5 | 1.82 |
| Sdev | 0.16 | 8.4 | 1.2 | 0.59 |

*^a^*Condition: Tested by uniaxial tensile tests. Strain rate of 10 mm/min, E: Tensile modulus. *σ*_b_: break strength.

# Preparation of quaternary ammonium materials

## Synthesis of tertiary amine alcohol (S-TAA)

Scheme S2. Synthesis of **S-TAA**: To a solution of **MBCA** (210 mg, 1 mmol) in 5 mL of anhydrous DCM was added glycidol (296 mg, 4 mmol). The resulting mixture was heated to 40 ℃ for overnight. After the concentration of solution, the residual product is the aim product **S-TAA**.

## Tertiary amine alcohol (D-TAA) from the depolymerization of epoxy resin

***Step***: Epoxy resin and methanol were added to a 250 mL flask, and the mixture was heated at 70 °C for two days. After concentrating the solution, the residue was dissolved in a mixed solvent (DCM/PE = 1:1). The mixture was cooled in a refrigerator overnight, and the clear supernatant was transferred to another flask. This procedure was repeated twice to ensure that no ester product remained. After concentrating the clear solution and the remaining residue, the ester products and the tertiary amine alcohol (**D-TAA**) were obtained, respectively.

#### Characterization of building block from **M2-MBCA**-based composite

Figure S55. ¹H NMR spectra (400 MHz, CDCl₃) of the esters recovered from the methanolysis-induced degradation of the **M2/MBCA** composite at 70 °C.

#### Characterization of S-TAA and D-TAA

^1^H-NMR of **S-TAA** and **D-TAA**

Figure S56. ^1^H-NMR (CD_3_OD) analysis of **S-TAA** and **D-TAA**. Both of them have the structures of tertiary amine alcohol. D-TAA has the residual aromatic structures from the epoxy resin.

ESI analysis of **S-TAA** and **D-TAA.**

Figure S57. ESI analysis of S-TAA and D-TAA. The detected peaks (blue and yellow points) indicate that both samples are mixtures of amine alcohols.

FT-IR analysis of **S-TAA** and **D-TAA**

Figure S58. FT-IR spectra of synthesized polyol **S-TAA** and methanolysis-derived polyol **D-TAA**. The blue-highlighted regions denote absorption bands assignable to residual aromatic structures, with possible contributions from amide-related vibrations.

## Synthesis and characterization of quaternary ammonium materials

#### Synthesis of quaternary ammonium materials

Scheme S3. Literature procedure^[3]^ was modified for the synthesis of quaternary ammonium materials.

***Step***: Equimolar amounts of the ditertiary amine (100 mg) and the corresponding dibromide (DBD, DBT, or DBO) were dissolved in DMF (4 mL) and stirred in a 10 mL vial at 80 °C for 24 h. Upon completion, the reaction mixture was poured into ethyl acetate to precipitate the quaternary ammonium product. The precipitate was collected and washed with ethyl acetate twice, then dried in a vacuum oven until complete removal of residual DMF.

#### Characterization of quaternary ammonium materials

#### ^1^H-NMR of quaternary ammonium materials

Figure S59. ^1^H NMR spectra (400 MHz, CD_3_OD) of quaternary ammonium materials. The marketed part (~7 ppm) is the residual aromatic structure, (2.5-3.0 ppm) peak is the terminal CH_2_-Br and N-CH_2_-R.

#### FT-IR analysis of quaternary ammonium materials

Figure S60. FT-IR spectra of quaternary ammonium materials. The marketed parts are the residual aromatic structures.

#### Zeta potential analysis of quaternary ammonium materials

Table S9 Zeta potential of different quaternary ammonium materials

| Name | ζ (mV) |
| --- | --- |
| **S-TAA** | -20.67±0.69 |
| **D-TAA** | -13.71±0.30 |
| **S-TAA-DBO** | +38.31±1.86 |
| **D-TAA-DBO** | +31.30±0.38 |
| **D-TAA-DBD** | +33.45±3.05 |
| **D-TAA-DBT** | +31.42±3.13 |

# DFT calculations Chapter

To investigate by quantum chemical means why the methanolysis is possible under mild conditions when the linear chains are connected to the phenyl ring via an ether oxygen, but not when they are connected via an alkyl carbon, we constructed the small model systems shown in Scheme S 4. **3_P(OA)_2_** (**p**hen**o**xy**a**cetate) is the base model system for the systems in which an ether oxygen connects the chain to the phenyl ring, while **4_P(A)_2_** (**p**henyl**a**cetate) is the base model system for the systems in which the connection is made through an alkyl carbon.

Scheme S4. Names for the model systems used in the computational investigation.

## Thermodynamics and kinetics

First, we calculated the thermodynamics of the methanolysis of **3_P(OA)_2_** or **4_P(A)_2_** to **9_POD** (**p**henylenebis**o**xy**d**iacetate) or **10_PD** (**p**henylene**d**iacetate) and **11_DA** (**d**imethyl**a**minopropanediol). The reactions are shown in Scheme S5.

Scheme S5.: Computationally investigated methanolysis of 3_P(OA)_2_ to 9_POD and 11_DA, and 4_P(A)_2_ to 10_PD and 11_DA, respectively.

The Gibbs energy of the reaction was calculated at the B3LYP-D4/def2-TZVPPD//PBE-D4/def2-SVP [COSMO(methanol)]-out level of theory (see computational details).^[4]^ For the methanolysis of **3_P(OA)_2_** a reaction Gibbs energy $\Delta_{R}G\left( 298.15 K \right)$ of –12.5 kJ mol^–1^ is found. The methanolysis of **4_P(A)_2_** under ambient conditions is thermodynamically slightly more favorable, with a $\Delta_{R}G\left( 298.15 K \right)$ of –18.9 kJ mol^–1^. Both reactions are thus exergonic and proceed spontaneously, that is, without requiring energy input from the outside. Since the reaction is carried out at 70 °C, $\Delta_{R}G\left( 368.15 K \right)$ was calculated as well, with $\Delta_{R}G\left( \boldsymbol{3\_P}\mathbf{(OA)}_{\boldsymbol{2}}\mathbf{,}368.15 K \right)$ being –17.2 kJ mol^–1^ and $\Delta_{R}G\left( \boldsymbol{4\_P(A)}_{\mathbf{2}},368.15 K \right)$ being –22.4 kJ mol^–1^. This shows that the reaction becomes thermodynamically more favorable at higher temperatures. It is notable that the difference in reaction Gibbs energy $\Delta_{R}G\left( T \right)$ between **3_P(OA)_2_** and $\boldsymbol{4\_P}\mathbf{(A)}_{\boldsymbol{2}}$ changes from –6.4 kJ mol^–1^ to –5.4 kJ mol^–1^. This is due to a disproportionately larger increase in entropy with temperature for **9_POD** compared to **10_PD**. The likely reason is that **9_POD** has more single bonds and is therefore more flexible, which leads to a pronounced increase in entropy with temperature. This flexibility is not present in $\boldsymbol{3\_P}\mathbf{(OA)}_{\boldsymbol{2}}$ as its two side chains are locked to each other by a hydrogen bond (see Figure S 3).
In the present model system and model methanolysis (Scheme S5) two molecules react to give two other molecules, so that entropic effects are small. In the actual polymer, by contrast, one molecule is split into many. These entropy-based effects will therefore be much more pronounced. This means that for both systems, those connected to the phenyl ring via an ether oxygen and those connected via an alkyl carbon, the methanolysis will become more exergonic with increasing temperature. Furthermore, the entropy increase will be even larger for the systems connected to the phenyl ring via an ether oxygen. Based on the reaction Gibbs energy $\Delta_{R}G\left( T \right)$ calculated here, it can be assumed that if the polymer is split into seven or more **9_POD/10_PD** molecules, the methanolysis becomes more exergonic for the systems connected to the phenyl ring via an ether oxygen.
Nevertheless, the calculations show that the methanolysis is thermodynamically favorable for both types of system, those connected to the phenyl ring via an ether oxygen and those connected via an alkyl carbon. In the experiment, the methanolysis is faster for the systems in which an ether oxygen is connected to the phenyl ring. Kinetic control must therefore be the reason for this experimental observation. Based on the Bell–Evans–Polanyi principle^[5]^ and supported by Hammond's postulate^[6]^ the activation energy is proportional to the reaction enthalpy $\Delta_{R}H\left( T \right)$ for structurally related chemical transformations. For the methanolysis (Scheme S5) of **3_P(OA)_2_** $\Delta_{R}H\left( 298.15 K \right)$ is 6.6 kJ mol^–1^ and $\Delta_{R}H\left( 368.15 K \right)$=6.4 kJ mol^–1^, while for **4_P(A)_2_** $\Delta_{R}H\left( 298.15 K \right)$ is –4.3 kJ mol^–1^ and $\Delta_{R}H\left( 368.15 K \right)$=–4.1 kJ mol^–1^. Thus, even at elevated temperatures **4_P(A)_2_** is enthalpically more favorable. It follows that, contrary to the experimental finding, the systems in which the linear chains are connected to the phenyl ring via an alkyl carbon should be more susceptible to methanolysis. For reaction energies, B3LYP has an accuracy of only 20-40 kJ mol^–1^,^[7]^ but for structurally closely related compounds such as those studied here the error is much smaller, and the trend is likely to be correct. However, the COSMO-out solvent model primarily accounts for electrostatic polarization. Specific short-range solvation effects such as hydrogen bonds are not considered. Wu et al.^[8]^ recently showed for similar systems that activation by hydrogen bonds plays a crucial role. This is therefore investigated in a separate step.

Figure S61: : Structure with important bond lengths and angles for **1_P(OA)**. The structure was optimized at the PBE-D4/def2-SVP [COSMO(methanol)] level of theory. Color code for the atoms: grey: carbon, red: oxygen, blue: nitrogen, white: hydrogen.

Figure S62: Structure with important bond lengths and angles for **2_P(A)**. The structure was optimized at the PBE-D4/def2-SVP [COSMO(methanol)] level of theory. Color code for the atoms: grey: carbon, red: oxygen, blue: nitrogen, white: hydrogen.

Figure S63: Structure with important bond lengths and angles for **3_P(OA)_2_**. The structure was optimized at the PBE-D4/def2-SVP [COSMO(methanol)] level of theory. Color code for the atoms: grey: carbon, red: oxygen, blue: nitrogen, white: hydrogen.

Figure S64: Structure with important bond lengths and angles for **4_P(A)_2_**. The structure was optimized at the PBE-D4/def2-SVP [COSMO(methanol)] level of theory. Color code for the atoms: grey: carbon, red: oxygen, blue: nitrogen, white: hydrogen.

Figure S65: Structure with important bond lengths and angles for **5_P(OA)_2_-RR**. The structure was optimized at the PBE-D4/def2-SVP [COSMO(methanol)] level of theory. Color code for the atoms: grey: carbon, red: oxygen, blue: nitrogen, white: hydrogen.

Figure S66: Structure with important bond lengths and angles for **5_P(OA)_2_-RS**. The structure was optimized at the PBE-D4/def2-SVP [COSMO(methanol)] level of theory. Color code for the atoms: grey: carbon, red: oxygen, blue: nitrogen, white: hydrogen.

Figure S67: Structure with important bond lengths and angles for **6_(P(A)_2_)_2_-RR**. The structure was optimized at the PBE-D4/def2-SVP [COSMO(methanol)] level of theory. Color code for the atoms: grey: carbon, red: oxygen, blue: nitrogen, white: hydrogen.

Figure S68: Structure with important bond lengths and angles for **6_(P(A)_2_)_2_-RS**. The structure was optimized at the PBE-D4/def2-SVP [COSMO(methanol)] level of theory. Color code for the atoms: grey: carbon, red: oxygen, blue: nitrogen, white: hydrogen.

Figure S69: Structure with important bond lengths and angles for **7_(P(OA)_2_)_2_-RR-MeOH** The structure was optimized at the PBE-D4/def2-SVP [COSMO(methanol)] level of theory. Color code for the atoms: grey: carbon, red: oxygen, blue: nitrogen, white: hydrogen.

Figure S70: Structure with important bond lengths and angles for **7_(P(OA)_2_)_2_-RS-MeOH**. The structure was optimized at the PBE-D4/def2-SVP [COSMO(methanol)] level of theory. Color code for the atoms: grey: carbon, red: oxygen, blue: nitrogen, white: hydrogen.

Figure S71: Structure with important bond lengths and angles for **8_(P(A)_2_)_2_-RR-MeOH**. The structure was optimized at the PBE-D4/def2-SVP [COSMO(methanol)] level of theory. Color code for the atoms: grey: carbon, red: oxygen, blue: nitrogen, white: hydrogen.

Figure S72: Structure with important bond lengths and angles for **8_(P(A)_2_)_2_-RS-MeOH**. The structure was optimized at the PBE-D4/def2-SVP [COSMO(methanol)] level of theory. Color code for the atoms: grey: carbon, red: oxygen, blue: nitrogen, white: hydrogen.

Figure S73: Structure with important bond lengths and angles for **9_POD**. The structure was optimized at the PBE-D4/def2-SVP [COSMO(methanol)] level of theory. Color code for the atoms: grey: carbon, red: oxygen, blue: nitrogen, white: hydrogen.

Figure S74: Structure with important bond lengths and angles for **10_PD**. The structure was optimized at the PBE-D4/def2-SVP [COSMO(methanol)] level of theory. Color code for the atoms: grey: carbon, red: oxygen, blue: nitrogen, white: hydrogen.

Figure S75: Structure with important bond lengths and angles for **11_DA**. The structure was optimized at the PBE-D4/def2-SVP [COSMO(methanol)] level of theory. Color code for the atoms: grey: carbon, red: oxygen, blue: nitrogen, white: hydrogen.

Figure S76: Structure with important bond lengths and angles for **methanol**. The structure was optimized at the PBE-D4/def2-SVP [COSMO(methanol)] level of theory. Color code for the atoms: grey: carbon, red: oxygen, blue: nitrogen, white: hydrogen.

## Methanol and carbonyl activation

The experimental results show that methanolysis proceeds substantially faster under mild conditions when the linear chains are connected to the phenyl ring through an ether oxygen, whereas it proceeds more slowly when the connection is made through an alkyl carbon. However, the quantum chemical analysis of the thermodynamics and kinetics of the methanolysis of the small model systems **3_P(OA)_2_** (connected to the phenyl ring via an ether oxygen) and $\boldsymbol{4\_P}\mathbf{(A)}_{\boldsymbol{2}}$(connected to the phenyl ring via an alkyl carbon) indicates that both have the same reactivity, or that the methanolysis is even more favorable for $\boldsymbol{4\_P}\mathbf{(A)}_{\boldsymbol{2}}$. This contradicts [C1.1] the experimental findings. However, the quantum chemical calculations did not take specific short-range solvation effects such as hydrogen bonds into account. Wu et al.^[8]^ showed that methanol and carbonyl activation are important for understanding the reactivity. For this analysis they used partial charges $\delta$ as a descriptor. Following their approach, partial charges are also used here to elucidate the reactivity. Figure S61 to Figure S76 show the final structures used in the present study and in the following discussion. **3_P(OA)_2_** (**p**hen**o**xy**a**cetate) is the base model system for the systems in which an ether oxygen connects the chain to the phenyl ring, while **4_P(A)_2_** (**p**henyl**a**cetate is the base model system for the systems in which the connection is made through an alkyl carbon. Removing one side chain from each of them leads to **1_P(OA)** and **2_P(A)**. These were originally the starting systems, but the side chains in **3_P(OA)_2_** (Figure S63) and **4_P(A)_2_** (Figure S64) showed moderate to strong hydrogen bonds (compare Table S 1) between the two side chains. These hydrogen bonds are important; therefore, **3_P(OA)_2_** and **4_P(A)_2_** were chosen as base systems.

Table S 10: Classification of hydrogen bonds. X–H···Y values taken from Steiner,^[9]^ who adapted them from Jeffrey.^[13]^

| **Hydrogen bond strength:** | **Strong** | **Moderate** | **Weak** |
| --- | --- | --- | --- |
| **H···Y distance** | 1.2 – 1.5 Å | 1.5 – 2.2 Å | >2.2 Å |
| **X···Y distance** | 2.2 – 2.5 Å | 2.5 – 3.2 Å | >3.2 Å |
| **X-H···Y angle** | 170° – 180° | 130° – 170° | 90° – 130° |

To obtain a description closer to the environment in the polymer, two molecules of **3_P(OA)_2_** or **4_P(A)_2_** were combined to form **5_(P(OA)_2_)_2_-RR/5_(P(OA)_2_)_2_-RS** or **6_(P(A)_2_)_2_-RR/6_(P(A)_2_)_2_-RS**. In these structures **3_P(OA)_2_** and **4_P(A)_2_** either both have the R configuration or one has the R and the other the S configuration. No calculation is required for the corresponding SS isomers, as their energies, partial charges, bond lengths and angles are in each case identical to those of the RR system. As a last step, one methanol molecule was added, resulting in **7_(P(OA)_2_)_2_-RR/RS-MeOH** and **8_(P(A)_2_)_2_-RR/RS-MeOH**, respectively. For all calculated systems (Scheme S4), the conformer with the lowest energy (i.e. the energetically most favorable one) is used for the analysis and shown in Figure S61 to Figure S76. The only exception is **7_(P(OA)_2_)_2_-RS-MeOH**. Here, the lowest-energy conformer with a methanol molecule adjacent to the carboxyl group (at about 3 Å distance and with an angle of 89–120°, as for the other systems) was chosen, so that methanol is in a position to initiate the methanolysis.
Table S11 shows the partial charge $\delta$ of the oxygen atom in methanol, both for free methanol and for the methanol bound in **7_(P(OA)_2_)_2_-RR/RS-MeOH** and **8_(P(A)_2_)_2_-RR/RS-MeOH**. From the difference in partial charge $\boldsymbol{\Delta}\delta$, it can be seen that for **7_(P(OA)_2_)_2_-RS-MeOH** the smallest activation (smallest reduction in partial charge) of the methanol oxygen is observed. The reason is that in this case the methanol (Figure S70) binds to the oxygen of an alkoxy group of the carboxylate group, whereas for **7_(P(OA)_2_)_2_-RR-MeOH** and **8_(P(A)_2_)_2_-RR/RS-MeOH** to an alkoxy group that is placed at the alkane chain. The largest activation (largest reduction in partial charge) is found for **7_(P(OA)_2_)_2_-RR-MeOH** (Figure S69). There the methanol oxygen binds not just to an alkoxy group, but to an alkoxy group that is itself activated by binding to another alkoxy group, which in turn is activated by binding to the carbonyl group of a carboxylate group. In the case of **8_(P(A)_2_)_2_-RR-MeOH** (Figure S 71) the methanol binds to an alkoxy group that has only two weak hydrogen bonds (Table S 10) with small angles and large bond distances, connecting it to an amino group and a distant carbonyl group of a carboxylate group. As a result, the partial charge on the methanol oxygen is reduced much less, and the methanol is therefore less activated. **8_(P(A)_2_)_2_-RS-MeOH** (Figure S 72) shows the same advantageous binding situation as **7_(P(OA)_2_)_2_-RR-MeOH**, with the methanol oxygen binding to an activated alkoxy group, which in turn binds to another alkoxy group that is itself activated by binding to the carbonyl group of a carboxylate group. Nevertheless, the methanol oxygen in **8_(P(A)_2_)_2_-RS-MeOH** is less activated (–0.029 ***e*** compared to –0.034 ***e***) than in **7_(P(OA)_2_)_2_-RR-MeOH**. A comparison of the individual hydrogen bonds shows that they are on average 0.071 Å longer in **8_(P(A)_2_)_2_-RS-MeOH** than in **7_(P(OA)_2_)_2_-RR-MeOH**. In addition, the bond angle of the second hydrogen bond in the chain (shown in green) in **8_(P(A)2)2-RS-MeOH** is 11.6° smaller than the smallest angle in **7_(P(OA)_2_)_2_-RR-MeOH**. The longer bonds and the smaller angle make the hydrogen bonds weaker ^[9-10]^, and thereby reduce the activation.

Table S11: Partial charges $\delta$ calculated with NPA at the B3LYP-D4/def2-TZVPPD//PBE-D4/def2-SVP [COSMO(methanol)] level of theory. The charges $\delta$ were calculated for the oxygen atom of methanol. The differences $\boldsymbol{\Delta}\delta$ are given between the partial charge $\delta$ of the oxygen atom of free, unbound methanol and that of the methanol in the other systems. The charges are given in elementary charge **e**.

| **System** | **Atom** | $\delta$ in ***e*** | $\boldsymbol{\Delta}\delta$ **to MeOH** in ***e*** |
| --- | --- | --- | --- |
| **MeOH** | MeOH oxygen | –0.752 | 0 |
| **7_(P(OA)_2_)_2_-RR-MeOH** | MeOH oxygen | –0.785 | –0.034 |
| **7_(P(OA)_2_)_2_-RS-MeOH** | MeOH oxygen | –0.759 | –0.008 |
| **8_(P(A)_2_)_2_-RR-MeOH** | MeOH oxygen | –0.778 | –0.026 |
| **8_(P(A)_2_)_2_-RS-MeOH** | MeOH oxygen | –0.780 | –0.029 |

Table S13 shows the partial charge $\delta$ of the carbon atom in the carboxylate group. Depending on whether a system is based on molecules of **3_P(OA)_2_** or **4_P(A)_2_**, either the one or the other is used as the reference for $\boldsymbol{\Delta}\boldsymbol{\delta}$. Overall, the values fluctuate considerably more than the partial charges of the methanol oxygen. The strongest activation (strongest increase in charge) for a carbon in the carboxylate is found in **5_(P(OA)_2_)_2_-RR** and **6_(P(A)_2_)_2_-RS**. In both cases the carboxylate groups form moderate to strong hydrogen bonds to alkoxy groups. Together with the partial charges for the methanol oxygen this shows how important hydrogen bond networks are for activating the active sites of the methanolysis.

Table S13: Partial charges $\delta$ calculated with NPA at the B3LYP-D4/def2-TZVPPD//PBE-D4/def2-SVP [COSMO(methanol)] level of theory. The charges $\delta$ were calculated for the carbon atom of the carboxylate group in the alkyl chain that contains the amine. Some systems contain two identical molecules that each have such a group; it is therefore indicated whether the group belongs to molecule number one or two. The differences $\boldsymbol{\Delta}\delta$ are given between the partial charge $\delta$ of the carbon atom in either of **3_P(OA)_2_** or **4_P(A)_2_** and that in the systems built from them. The charges are given in elementary charge **e**.

| **System** | **Atom** | **molecule** | $\boldsymbol{\delta}$ **in *e*** | $\boldsymbol{\Delta}\boldsymbol{\delta}$ **to 3_P(OA)_2_/4_P(A)_2_ in *e*** |
| --- | --- | --- | --- | --- |
| 1_P(OA) | Carbonyl carbon | 1 | 0.763 | –0.003 |
| 2_P(A) | Carbonyl carbon | 1 | 0.805 | 0.003 |
| 3_P(OA)_2_ | Carbonyl carbon | 1 | 0.766 | 0 |
| 4_P(A)_2_ | Carbonyl carbon | 1 | 0.802 | 0 |
| 5_(P(OA)_2_)_2_-RR | Carbonyl carbon | 1 | 0.773 | 0.007 |
| 5_(P(OA)_2_)_2_-RR | Carbonyl carbon | 2 | 0.787 | 0.021 |
| 5_(P(OA)_2_)_2_-RS | Carbonyl carbon | 1 | 0.765 | –0.001 |
| 5_(P(OA)_2_)_2_-RS | Carbonyl carbon | 2 | 0.766 | 0.000 |
| 6_(P(A)_2_)_2_-RR | Carbonyl carbon | 1 | 0.810 | 0.007 |
| 6_(P(A)_2_)_2_-RR | Carbonyl carbon | 2 | 0.812 | 0.009 |
| 6_(P(A)_2_)_2_-RS | Carbonyl carbon | 1 | 0.818 | 0.016 |
| 6_(P(A)_2_)_2_-RS | Carbonyl carbon | 2 | 0.829 | 0.027 |
| 7_(P(OA)_2_)_2_-RR-MeOH | Carbonyl carbon | 1 | 0.766 | 0.000 |
| 7_(P(OA)_2_)_2_-RR-MeOH | Carbonyl carbon | 2 | 0.767 | 0.001 |
| 7_(P(OA)_2_)_2_-RS-MeOH | Carbonyl carbon | 1 | 0.775 | 0.009 |
| 7_(P(OA)_2_)_2_-RS-MeOH | Carbonyl carbon | 2 | 0.767 | 0.001 |
| 8_(P(A)_2_)_2_-RR-MeOH | Carbonyl carbon | 1 | 0.821 | 0.019 |
| 8_(P(A)_2_)_2_-RR-MeOH | Carbonyl carbon | 2 | 0.805 | 0.003 |
| 8_(P(A)_2_)_2_-RS-MeOH | Carbonyl carbon | 1 | 0.821 | 0.019 |
| 8_(P(A)_2_)_2_-RS-MeOH | Carbonyl carbon | 2 | 0.803 | 0.001 |
| 9_POD | Carbonyl carbon | 1 | 0.757 | –0.008 |
| 10_PD | Carbonyl carbon | 1 | 0.798 | –0.005 |

## Summary and conclusion of the theoretical results

The thermodynamic and kinetic analyses based on the smaller model systems, in which specific short-range solvation effects such as explicit hydrogen-bonding interactions were not considered, do not fully explain the experimentally observed enhancement in methanolysis for the ether-containing structures. However, when a larger model system consisting of two substrate molecules and one methanol molecule was considered, more complex intermolecular hydrogen-bonding arrangements became accessible. These local interaction networks may influence the polarization and positioning of methanol and facilitate its reactive approach toward the ester carbonyl.

In the ether-containing systems, the ester functionality is connected to the rigid phenyl ring through a more flexible ether-containing linkage and is therefore located farther from the aromatic framework. The presence of additional rotatable single bonds increases the conformational freedom of the side chains, which may improve the accessibility of the ester functionality to methanol and facilitate the formation of favorable local hydrogen-bonding arrangements around the reaction center. These effects may jointly influence methanol polarization, molecular orientation, and the reactive arrangement between methanol and the ester carbonyl.

Taken together, the calculations suggest that the enhanced methanolysis of the ether-containing structures may originate from their increased conformational flexibility and accessibility, which enable more favorable local interaction environments around the ester functionality. The exact hydrogen-bonding arrangement depends on the local conformation and stereochemical configuration. Therefore, the present calculations provide a plausible local-structure-based explanation for the experimental observations, rather than evidence for a single universally strengthened hydrogen bond.11

# Computational details

## Structure optimization and calculation of Gibbs energies

To identify the energetically most favorable conformers and the equilibrium structures of the molecular systems of interest, automated conformer searches were performed with CREST 2.12.^[11]^ In the first step the iterative metadynamics in conjunction with a genetic structure crossing (iMTD-GC) algorithm was employed. Afterwards, the extended tight-binding model GFN2-xTB was applied to perform structure optimizations.^[12]^ To simulate methanol, the solvent used in the experimental investigation, the implicit analytical linearized Poisson–Boltzmann (ALPB)^[13]^ solvent model was employed. The energy threshold was set to 104.6 kJ mol^–1^ for all systems except **7_(P(OA)_2_)_2_-RR/RS-MeOH** and **8_(P(A)_2_)_2_-RR/RS-MeOH**. Due to the stark increase of computational demand with system size, the threshold was set to 52.3 kJ mol^–1^ for these four systems. However, this is still more than twice as high as the rotational barrier of an alkane such as butane, which is 23.1 kJ mol^–1^.^[14]^
Since the conformational search did not give consistent results for the larger systems, it was performed several times with different starting structures; the corresponding numbers are given in Table S14. The construction of the starting structures was generally based on chemical intuition. In the case of **7_(P(OA)_2_)_2_-RR/RS-MeOH** and **8_(P(A)_2_)_2_-RR/RS-MeOH** starting structures for the conformer search with CREST were based on the three most favorable structures found for **5_P(OA)_2_-RR/RS** and **6_P(A)_2_-RR/RS** at the density functional theory (DFT) level.
The geometry optimizations in the CREST conformer search were performed with tight^[15]^ convergence thresholds (of 10^–6^ Hartree for the energy and 8∙10^–4^ Hartree per approximate normal coordinate (ANC) α for the root-mean-square magnitude of the potential energy gradient with respect to atomic coordinates). Because of these tight criteria, only one conformer was obtained from each individual conformer search; for example, for **6_(P(A)_2_)_2_-RR,** 33 different conformer searches with different starting conformers were carried out, which resulted in 33 different conformers.

Table S14: Number of conformers used as input for the conformer searches with CREST.

| **System** | **Number of Conformers** |
| --- | --- |
| **1_P(OA)** | 4 |
| **2_P(A)** | 4 |
| **3_P(OA)_2_** | 8 |
| **4_P(A)_2_** | 8 |
| **5_(P(OA)_2_)_2_-RR** | 33 |
| **5_(P(OA)_2_)_2_-RS** | 36 |
| **6_(P(A)_2_)_2_-RR** | 33 |
| **6_(P(A)_2_)_2_-RS** | 36 |
| **7_(P(OA)_2_)_2_-RR-MeOH** | 18 |
| **7_(P(OA)_2_)_2_-RS-MeOH** | 18 |
| **8_(P(A)_2_)_2_-RR-MeOH** | 18 |
| **8_(P(A)_2_)_2_-RS-MeOH** | 18 |
| **9_POD** | 5 |
| **10_PD** | 5 |
| **11_DA** | 5 |
| **MeOH** | 1 |

The structures found with CREST were assessed with DFT. To increase the number of investigated conformers, the starting structures used for the CREST calculations were also used for the screening on the DFT level in the case of the more complex systems, i.e. **5_P(OA)_2_-RR/RS**, **6_P(A)_2_-RR/RS**, **7_(P(OA)_2_)_2_-RR/RS-MeOH** and **8_(P(A)_2_)_2_-RR/RS-MeOH**. This procedure was also used for **9_POD**, **10_PD**, **11_DA**. The DFT calculations were carried out with the software package Turbomole 7.6.1.^[16]^ For all calculations, the Resolution-of-Identity (RI) approximation^[1f, 1g, 1o]^ and the multiple grid m4 were utilized.^[17]^ The solvent, methanol, was simulated with the conductor-like screening model COSMO.^[18]^ Thereby, methanol was described by its relative permittivity $\varepsilon_{r}=32.613$ and its refractive index $n_{D}=1.3288$.^[19]^ The selected conformers were optimized at the PBE-D4/def2-SVP [COSMO(methanol)] level of theory,^[4a-i]^ with convergence thresholds for the energy in the self-consistent field cycle (SCF) of 10^-6^ Hartree and for the gradients of 10^–3^ atomic units. The most favorable conformer of each system was optimized further, with convergence thresholds of 10^–8^ Hartree and 10^–4^ atomic units for the energy and the gradients, respectively. Afterwards, vibrational frequency analyses were carried out for these systems. They were performed at the same level of theory utilizing the Turbomole NumForce script and also accounted for the fast contribution of the solvent.^[20]^ In all cases the structures were confirmed to be energetic minima, as they proved to have no imaginary frequencies. In In addition, these calculations provide thermal contributions $G_{T}^{ro-vib}$ to the Gibbs energy of these systems, dependent on the temperature $T$.

As a final step, single-point calculations were carried out at the B3LYP-D4/def2-TZVPPD [COSMO(methanol)]-out^[4a, 4b, 4j-p]^ level of theory. The “-out” signifies here the use of the outlying charge correction^[4p]^ for the calculation of the final electronic energy $E_{\mathrm{elec}}$. The convergence threshold for the SCF energy was set to 10^–6^ Hartree. The electronic energies at the B3LYP-D4/def2-TZVPPD [COSMO(methanol)]-out level of theory were used in conjunction with the thermal contributions $G_{T}^{ro-vib}$ at the PBE-D4/def2-SVP [COSMO(methanol)] level of theory to obtain the Gibbs energy of the systems $G^{\circ}\left( T \right)$:

$$G^{\circ}\left( T \right)=E_{\mathrm{elec}}+G_{T}^{ro-vib}+G_{conc,T}^{\mathrm{corr}} .$$

(1)

This includes the temperature $T$ dependent correction term $G_{conc,T}^{\mathrm{corr}}$. The term is used to adjust the concentration of all systems to 1 mol L^–1^ at a pressure of 101325 Pa. The only exception is methanol, as it is used as the solvent. Based on its density^[18]^ the concentration of methanol in methanol is 24.548 mol L^–1^ at a pressure of 101325 Pa and 298.15 K. $G_{conc,T}^{\mathrm{corr}}$ is calculated with

$$G_{\mathrm{conc}}^{\mathrm{corr}}=-RT\cdot\ln\left( \frac{V_{m}^{\mathrm{new}}}{V_{m}^{\mathrm{old}}} \right)$$

(2)

where $R$ is the molar gas constant, and $V_{m}^{\mathrm{new}}$ [$=0.001 m^{3} \mathrm{mol}^{-1} (\mathrm{and}0.000041 m^{3} \mathrm{mol}^{-1} for methanol)$] the new volume, and $V_{m}^{\mathrm{old}}[=0.024465 m^{3} \mathrm{mol}^{-1}(at 298.15 K) and 0.030209 m^{3} \mathrm{mol}^{-1}(at 368.15 K),]$ the old molar volume.

We would like to add that the def2-QZVP basis set would have been the better choice, because in DFT it already reaches chemical accuracy, unlike def2-TZVPPD,^[22]^ and B3LYP-D4/def2-QZVP [COSMO(methanol)]-out required less computational time in a test calculation for **7_(P(OA)_2_)_2_-RR/RS-MeOH**. However, in order to obtain partial charges comparable with those of the investigation by Wu et al.^[8]^ B3LYP-D4/def2-TZVPPD was applied here. Unlike Wu et al.^[5]^ a solvent model, [COSMO(methanol)]-out, was used here to take the effects of the environment into account. In line with Wu et al.^[8]^ the partial charges here are calculated with a natural population analysis (NPA), in the present study at the B3LYP-D4/def2-TZVPPD//PBE-D4/def2-SVP [COSMO(methanol)] level of theory.^[23]^

Table S15. NBO natural charges of selected atoms in the ether-containing and ether-free model systems.

| System | Model description | Configuration | Carbonyl C charge / e | Carbonyl O charge / e | Alkoxy O charge / e | Amine N charge / e | MeOH O charge / e |
| --- | --- | --- | --- | --- | --- | --- | --- |
| **POA** | Ether-containing single-molecule model | R | 0.76320 | -0.62401 | -0.75884 | -0.46507 |  |
| **PA** | Ether-free single-molecule model | R | 0.80523 | -0.63165 | -0.75785 | -0.46331 |  |
| **P-OA2** | Ether-containing single-molecule model | R | 0.76595 | -0.62706 | -0.76738 | -0.44611 |  |
| **P-A2** | Ether-free single-molecule model | R | 0.80242 | -0.63067 | -0.76366 | -0.44244 |  |
| **2P-OA2** | Ether-containing two-molecule model | RR | 0.77988 | -0.64370 | -0.76258 | -0.44384 |  |
| **2P-OA2** | Ether-containing two-molecule model | RS | 0.76562 | -0.62700 | -0.75745 | -0.45603 |  |
| **2P-A2** | Ether-free two-molecule model | RR | 0.81077 | -0.63108 | -0.77124 | -0.45346 |  |
| **2P-A2** | Ether-free two-molecule model | RS | 0.82379 | -0.64250 | -0.76662 | -0.44266 |  |
| **2P-OA2 + MeOH** | Ether-containing two-molecule model with MeOH | RR | 0.76637 | -0.61485 | -0.77451 | -0.44608 | -0.78511 |
| **2P-OA2 + MeOH** | Ether-containing two-molecule model with MeOH | RS | 0.77017 | -0.63127 | -0.75709 | -0.45644 | -0.75914 |
| **2P-A2 + MeOH** | Ether-free two-molecule model with MeOH | RR | 0.81331 | -0.63327 | -0.76696 | -0.45919 | -0.77787 |
| **2P-A2 + MeOH** | Ether-free two-molecule model with MeOH | RS | 0.81234 | -0.63431 | -0.77540 | -0.44092 | -0.78009 |
| **MeOH** | Free methanol | — |  |  |  |  | -0.75158 |

Note: For two-molecule models, the reported charges are arithmetic means of the corresponding atoms in the two molecular units.

Geometry optimizations were performed at the PBE-D4/def2-SVP level. NBO natural charges were calculated at the B3LYP-D4/def2-TZVPPD level using COSMO-out with MeOH as solvent.

# References

1.	Drevermann, B.; Lingham, A. R.; Hügel, H. M.; Marriott, P. J. Synthesis of Benzodioxepinone Analogues via a Novel Synthetic Route with Qualitative Olfactory Evaluation. *Helv. Chim. Acta* **2007**, *90*, 1006-1027.

2.	Wang, X.; Wei, T.; Chen, J.; Li, J. Phase Transfer Catalyzed Synthesis of N,N'-Diaryl-1,2-phenylene Dioxydiacetamides. *Synth. Commun.* **1996**, *26*, 2765-2773.

3.	Mattheis, C.; Zheng, M.; Agarwal, S. Closing One of the Last Gaps in Polyionene Compositions: Alkyloxyethylammonium Ionenes as Fast-Acting Biocides. *Macromol. Biosci.* **2012**, *12*, 341-349.

4.	aP. A. M. Dirac, *Proceedings of the Royal Society of London. Series A, Containing Papers of a Mathematical and Physical Character* **1929**, *123*, 714-733; bJ. C. Slater, *Physical review* **1951**, *81*, 385; cJ. P. Perdew, Y. Wang, *Physical Review B* **1992**, *45*, 13244; dJ. P. Perdew, K. Burke, M. Ernzerhof, *Physical review letters* **1996**, *77*, 3865; eF. Weigend, R. Ahlrichs, *Physical Chemistry Chemical Physics* **2005**, *7*, 3297-3305; fK. Eichkorn, O. Treutler, H. Öhm, M. Häser, R. Ahlrichs, *Chemical physics letters* **1995**, *240*, 283-290; gK. Eichkorn, O. Treutler, H. Oehm, M. Häser, R. Ahlrichs, *Chemical Physics* **1995**, *242*, 652-660; hE. Caldeweyher, C. Bannwarth, S. Grimme, *The Journal of chemical physics* **2017**, *147*, 034112; iE. Caldeweyher, S. Ehlert, A. Hansen, H. Neugebauer, S. Spicher, C. Bannwarth, S. Grimme, *The Journal of chemical physics* **2019**, *150*, 154122; jS. H. Vosko, L. Wilk, M. Nusair, *Canadian Journal of physics* **1980**, *58*, 1200-1211; kA. D. Becke, *Physical review A* **1988**, *38*, 3098; lC. Lee, W. Yang, R. G. Parr, *Physical review B* **1988**, *37*, 785; mA. D. Becke, *The Journal of Chemical Physics* **1993**, *98*, 5648-5652; nD. Rappoport, F. Furche, *The Journal of chemical physics* **2010**, *133*, 134105; oF. Weigend, *Physical chemistry chemical physics* **2006**, *8*, 1057-1065; pA. Klamt, V. Jonas, *The Journal of chemical physics* **1996**, *105*, 9972-9981.

5.	R. P. Bell, *Proceedings of the Royal Society of London. A. Mathematical and Physical Sciences* **1936**, *154*, 414-429.

6.	G. S. Hammond, *Journal of the American Chemical Society* **1955**, *77*, 334-338.

7.	aM. Bursch, J. M. Mewes, A. Hansen, S. Grimme, *Angewandte Chemie International Edition* **2022**, *61*, e202205735; bS. Grimme, A. Hansen, S. Ehlert, J.-M. Mewes, *The Journal of Chemical Physics* **2021**, *154*.

8.	X. Wu, P. Hartmann, D. Berne, M. De bruyn, F. Cuminet, Z. Wang, J. M. Zechner, A. D. Boese, V. Placet, S. Caillol, K. Barta, *Science* **2024**, *384*, eadj9989.

9.	T. Steiner, *Angewandte Chemie International Edition* **2002**, *41*, 48-76.

10.	G. A. Jeffrey, G. A. Jeffrey, *An introduction to hydrogen bonding, Vol. 12*, Oxford university press New York, **1997**.

11.	aP. Pracht, F. Bohle, S. Grimme, *Physical Chemistry Chemical Physics* **2020**, *22*, 7169-7192; bP. Pracht, S. Grimme, *Chemical Science* **2021**, *12*, 6551-6568; cS. Grimme, *Journal of chemical theory and computation* **2019**, *15*, 2847-2862.

12.	aC. Bannwarth, S. Ehlert, S. Grimme, *Journal of chemical theory and computation* **2019**, *15*, 1652-1671; bS. Grimme, C. Bannwarth, P. Shushkov, *Journal of chemical theory and computation* **2017**, *13*, 1989-2009; cP. Pracht, E. Caldeweyher, S. Ehlert, S. Grimme, *ChemRxiv* **2019**, 1-19.

13.	S. Ehlert, M. Stahn, S. Spicher, S. Grimme, *Journal of Chemical Theory and Computation* **2021**, *17*, 4250-4261.

14.	N. L. Allinger, J. T. Fermann, W. D. Allen, H. F. Schaefer III, *The Journal of Chemical Physics* **1997**, *106*, 5143-5150.

115.	P. Pracht, S. Grimme, C. Bannwarth, F. Bohle, S. Ehlert, G. Feldmann, J. Gorges, M. Müller, T. Neudecker, C. Plett, S. Spicher, P. Steinbach, P. A. Wesołowski, F. Zeller, *The Journal of Chemical Physics* **2024**, *160*.

16.	a*TURBOMOLE V7.6.1 2022, a development of University of Karlsruhe and Forschungszentrum Karlsruhe Gmbh, 1989-2007, TURBOMOLE GmbH, since 2007; available from* [*http://www.turbomole.com*](http://www.turbomole.com); bR. Ahlrichs, M. Bär, M. Häser, H. Horn, C. Kölmel, *Chemical Physics Letters* **1989**, *162*, 165-169; cS. G. Balasubramani, G. P. Chen, S. Coriani, M. Diedenhofen, M. S. Frank, Y. J. Franzke, F. Furche, R. Grotjahn, M. E. Harding, C. Hättig, A. Hellweg, B. Helmich-Paris, C. Holzer, U. Huniar, M. Kaupp, A. M. Khah, S. K. Khani, T. Müller, F. Mack, B. D. Nguyen, S. M. Parker, E. Perlt, D. Rappoport, K. Reiter, S. Roy, M. Rückert, G. Schmitz, M. Sierka, E. Tapavicza, D. P. Tew, C. v. Wüllen, V. K. Voora, F. Weigend, A. Wodyński, J. M. Yu, *The Journal of Chemical Physics* **2020**, *152*, 184107.

17.	K. Eichkorn, F. Weigend, O. Treutler, R. Ahlrichs, *Theoretical Chemistry Accounts* **1997**, *97*, 119-124.

18.	aA. Schäfer, A. Klamt, D. Sattel, J. C. Lohrenz, F. Eckert, *Physical Chemistry Chemical Physics* **2000**, *2*, 2187-2193; bA. Klamt, G. Schüürmann, *Journal of the Chemical Society, Perkin Transactions 2* **1993**, 799-805.

19.	W. M. Haynes (ed.), D. R. Lide (ed.), T. J. Bruno (ed.), *CRC Handbook of Chemistry and Physics*, 97 ed., CRC press, **2017**.

20.	aP. Deglmann, F. Furche, *The Journal of chemical physics* **2002**, *117*, 9535-9538; bP. Deglmann, F. Furche, R. Ahlrichs, *Chemical physics letters* **2002**, *362*, 511-518.

21.	M. J. O'Neil, *The Merck index: an encyclopedia of chemicals, drugs, and biologicals*, RSc Publishing, **2013**.

22.	F. Weigend, F. Furche, R. Ahlrichs, *The Journal of chemical physics* **2003**, *119*, 12753-12762.

23.	A. E. Reed, R. B. Weinstock, F. Weinhold, *The Journal of Chemical Physics* **1985**, *83*, 735-746.
